# Explanation of Fitts’ law in Reaching Movement based on Human Arm Dynamics

**DOI:** 10.1038/s41598-019-56016-7

**Published:** 2019-12-24

**Authors:** Misaki Takeda, Takanori Sato, Hisashi Saito, Hiroshi Iwasaki, Isao Nambu, Yasuhiro Wada

**Affiliations:** 1 Graduate School of Engineering, Nagaoka University of Technology, Nagaoka, Niigata 940-2188 Japan; 2 National Institute of Technology, Akita College, Akita, Akita 011-8511 Japan

**Keywords:** Biophysical models, Dynamical systems

## Abstract

Why does Fitts’ law fit various human behavioural data well even though it is not a model based on human physical dynamics? To clarify this, we derived the relationships among the factors applied in Fitts’ law—movement duration and spatial endpoint error—based on a multi-joint forward- and inverse-dynamics models in the presence of signal-dependent noise. As a result, the relationship between them was modelled as an inverse proportion. To validate whether the endpoint error calculated by the model can represent the endpoint error of actual movements, we conducted a behavioural experiment in which centre-out reaching movements were performed under temporal constraints in four directions using the shoulder and elbow joints. The result showed that the distributions of model endpoint error closely expressed the observed endpoint error distributions. Furthermore, the model was found to be nearly consistent with Fitts’ law. Further analysis revealed that the coefficients of Fitts’ law could be expressed by arm dynamics and signal-dependent noise parameters. Consequently, our answer to the question above is: Fitts’ law for reaching movements can be expressed based on human arm dynamics; thus, Fitts’ law closely fits human’s behavioural data under various conditions.

## Introduction

Speed-accuracy trade-offs (SATs), such as that between movement speed and spatial accuracy, are one of the most common phenomena in human movement. SATs have been actively studied since Woodworth^1^, who measured the accuracy of voluntary movement. Fitts’ law, the most famous SAT model, has been widely accepted in the field of Ergonomics and Human-Computer Interaction. Fitts^2^ applied Shannon’s information theory to the human motor system and proposed an empirical model that relates movement duration to movement distance and target width, called Fitts’ law, based on the following equation:

1 $$D=a+b\,{\log }_{2}(\frac{2A}{W})=a+b\,{I}_{d},$$

where *D* and *A* are the movement duration and distance, respectively, *W* is the target width, *I*_*d*_ is a logarithmic term in *W* and *A* called the index of difficulty, and *a* and *b* are intercept and slope in the linear equation, respectively, which are obtained as regression coefficients using *D* as an objective variable and *I*_*d*_ as an explanatory variable. Equation (1) can be rewritten as follows:

2 $$D=a^{\prime} +b\,{\log }_{2}(\frac{1}{W}),\,a^{\prime} =a+b({\log }_{2}A+1),$$

to emphasise that increasing the spatial accuracy required (i.e., decreasing *W*) at a given distance increases movement duration, and vice versa. Thus, Fitts’ law expresses a linear SAT function represented by features *a* and *b*, i.e., intercept and slope. Previous studies have already shown that Fitts’ law can be applied to behavioural data in a variety of contexts (reviewed in^3–5^). There is also a considerable body of research supporting the applicability of Fitts’ law to arm movements such as reciprocal tapping^2^, reaching movements^6^, and one-dimensional linear and two-dimensional planar arm movements^7–10^.

As mentioned above, Fitts’ law is a model that has been supported by many researchers for more than half a century, but the question as to how Fitts’ law can explain human movement under any condition, despite being an empirical model, remains unanswered. From a physical perspective, human movements are modelled by the fundamental law of motion, that is, using dynamics (including kinematics). Therefore, we hypothesised that Fitts’ law in reaching movement could be determined in terms of human arm dynamics. It is important to clarify the relationship between speed-accuracy and arm dynamics because it enables us to understand human motor performance at a more profound level.

There have been interesting findings regarding the slope of Fitts’ law, including demonstrations that the slope increases as the size of effectors^11^, the slope in elderly subjects is greater than that in young adults^12–14^, and the slope can be reduced with practice^15^. From these experimental facts, it is quite natural to consider that the coefficients of Fitts’ law are related to the physical dynamics factors. Recent studies have further shown that it is essential to investigate dynamics factors. For example, Hoffmann and Hui^16^ investigated the duration taken to move a given distance using different arm components such as fingers, wrists, forearms and the full arm and concluded that the moments of inertia and muscle torque strength of these components affect movement duration. Bertucco *et al*.^17^ showed that movements over a long distance were associated with Coriolis forces and had a larger intercept of Fitts’ law than those at short distances. Although these studies showed that dynamics factors such as arm segmentation and torque are involved in the SAT profiles of Fitts’ law, no sufficient theoretical explanation was provided. According to dynamical optimisation models such as the minimum (commanded) torque change criterion^18,19^, increasing movement duration changes the proportions of the respective torque components (inertial, Coriolis, centrifugal and viscous forces) along with the movement trajectory. To better understand the SAT mechanism in human arm movement, it is therefore important to construct a model that considers the contribution of these arm dynamics factors to movement.

Here, we derive the relationship between movement duration *D* and spatial endpoint error *W*—the factors of Fitts’ law—based on multi-joint forward- and inverse-dynamics models. As some researchers have pointed out that modelling the SAT requires consideration of the signal-dependent biological noise in the nervous system^20–22^, we consider modelling in the presence of signal-dependent noise. The *D* − *W* relationship that we derive nearly covers Fitts’ law. Furthermore, we theoretically demonstrate that arm dynamics and noise factors can affect both the slope and intercept of Fitts’ law.

## Explanation of Fitts’ Law Based on Human Arm Dynamics

**Figure 1:**
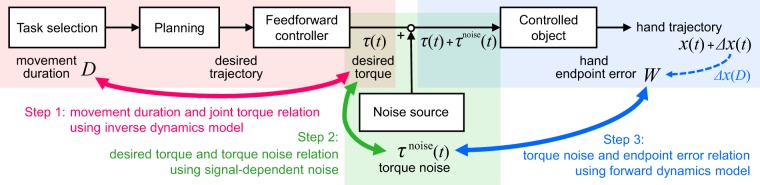
Schematic of modelling, involving feedforward control of rapid arm-reaching movement with movement planning and execution performed sequentially. The movement duration *D* is determined in the task selection stage. The desired trajectory is planned in the planning stage, in which the desired torque *τ*(*t*) at time *t*, which corresponds to the command signal, is generated by the feedforward controller implementing an inverse dynamics model. Finally, the torque noise *τ*^noise^(*t*) is added at the movement execution stage. The hand trajectory is generated by the controlled object (i.e., the arm), which implements a forward dynamics model using the noise-added torque *τ*(*t*) + *τ*^noise^(*t*). The result of converting the torque noise into the task space is the spatial error of the movement, and its endpoint *t* = *D* is the hand endpoint error.

To formulate the relationship between movement duration *D* and hand endpoint error *W* based on human arm dynamics, we assumed the feedforward control model shown in Fig. 1, and modelled the relationship between *D* and *W* using the following three steps: first, we derived the relationship between movement duration *D* and joint torque *τ* based on an inverse dynamics model; next, we derived the relationship between *τ* and torque noise *τ*^noise^ by defining a signal-dependent noise; finally, we derived the relationship between *τ*^noise^ and hand endpoint error *W* using a forward dynamics model. We then used the resulting *D* − *W* relation to represent Fitts’ law.

### Step 1: Determining the relationship between movement duration and joint torque

The general form of multi-joint (*N*-link) human arm dynamics on the horizontal plane can be modelled as a second-order nonlinear differential equation^18^:

3 $${\boldsymbol{\tau }}(t)={\boldsymbol{M}}({\boldsymbol{\theta }}(t))\mathop{{\boldsymbol{\theta }}}\limits^{{\boldsymbol{..}}}(t)+{{\boldsymbol{h}}}_{1}({\boldsymbol{\theta }}(t))[\dot{{\boldsymbol{\theta }}}(t)\dot{{\boldsymbol{\theta }}}(t)]+{{\boldsymbol{h}}}_{2}({\boldsymbol{\theta }}(t))[\dot{{\boldsymbol{\theta }}}{(t)}^{2}]+{\boldsymbol{B}}\dot{{\boldsymbol{\theta }}}(t),$$

where *t* represents time, $${\boldsymbol{\tau }}(t)={[{\tau }_{1}(t),{\tau }_{2}(t),\cdots ,{\tau }_{i}(t),\cdots ,{\tau }_{N}(t)]}^{{\rm{T}}}$$ is the joint torque, $${\boldsymbol{\theta }}(t)=[{\theta }_{1}(t),{\theta }_{2}(t),\cdots ,$$$${{\theta }_{i}(t),\cdots ,{\theta }_{N}(t)]}^{{\rm{T}}}$$ is the joint angular position, $$\dot{{\boldsymbol{\theta }}}(t)$$ and $$\dot{{\boldsymbol{\theta }}}(t)$$ are the joint angular velocity and acceleration, respectively, the superscript T denotes the transpose of a vector or matrix, and the subscript *i* denotes the joint number. The first term is the inertial force given in terms of the inertia matrix $${\boldsymbol{M}}({\boldsymbol{\theta }}(t))\in {{\mathbb{R}}}^{N\times N}$$. The second term is the Coriolis force, which is given in terms of a Coriolis force coefficient matrix $${{\boldsymbol{h}}}_{1}({\boldsymbol{\theta }}(t))\in {{\mathbb{R}}}^{N\times N(N-1)/2}$$. The third and fourth terms are the centrifugal and viscous forces in terms of the coefficient matrices of centrifugal and viscous forces, $${{\boldsymbol{h}}}_{2}({\boldsymbol{\theta }}(t))\in {{\mathbb{R}}}^{N\times N}$$ and $${\boldsymbol{B}}\in {{\mathbb{R}}}^{N\times N}$$, respectively. Further, $$[\dot{{\boldsymbol{\theta }}}(t)\dot{{\boldsymbol{\theta }}}(t)]$$ and $$[\dot{{\boldsymbol{\theta }}}{(t)}^{2}]$$ are the vectors in Coriolis and centrifugal forces, $${[{\dot{\theta }}_{1}(t){\dot{\theta }}_{2}(t),{\dot{\theta }}_{1}(t){\dot{\theta }}_{3}(t),\cdots ,{\dot{\theta }}_{N-1}(t){\dot{\theta }}_{N}(t)]}^{{\rm{T}}}$$ and $${[{\dot{\theta }}_{1}{(t)}^{2},{\dot{\theta }}_{2}{(t)}^{2},\cdots ,{\dot{\theta }}_{N}{(t)}^{2}]}^{{\rm{T}}}$$, respectively.

As the hand path observed in human arm movement is nearly invariant within a specific range of movement duration^23^, the following time-normalisation can be assumed^24^ (for a validation of the time-normalisation, see Discussion and Supplementary Fig. S1):

4 $$t=Ds,\,0\le t\le D\,(0\le s\le 1),\,{\theta }_{i}(t)={\theta }_{i}(Ds)={\tilde{\theta }}_{i}(s),$$

where *s* is the normalised time. Equation (3) can be further expressed in terms of the movement duration *D* as

5 $$\begin{array}{rcl}{\boldsymbol{\tau }}(t) & = & {\boldsymbol{\tau }}(Ds)=\frac{1}{{D}^{2}}\{{\boldsymbol{M}}(\tilde{{\boldsymbol{\theta }}}(s))\mathop{\tilde{{\boldsymbol{\theta }}}}\limits^{{\boldsymbol{..}}}(s)+{{\boldsymbol{h}}}_{1}(\tilde{{\boldsymbol{\theta }}}(s))[\dot{\tilde{{\boldsymbol{\theta }}}}(s)\dot{\tilde{{\boldsymbol{\theta }}}}(s)]\\  &  & +\,{{\boldsymbol{h}}}_{2}(\tilde{{\boldsymbol{\theta }}}(s))[\dot{\tilde{{\boldsymbol{\theta }}}}{(s)}^{2}]\}+\frac{1}{D}{\boldsymbol{B}}\dot{\tilde{{\boldsymbol{\theta }}}}(s).\end{array}$$

We can further assume that the approximation $1/D\simeq \lambda /{D}^{2}$ is possible over the general movement duration range, where *λ* is the coefficient of approximation (see Supplementary Fig. S2), giving:

6 $$\begin{array}{c}{\boldsymbol{\tau }}(Ds)\simeq \frac{1}{{D}^{2}}\{{\boldsymbol{M}}(\tilde{{\boldsymbol{\theta }}}(s))\mathop{\tilde{{\boldsymbol{\theta }}}}\limits^{{\boldsymbol{..}}}(s)+{{\boldsymbol{h}}}_{1}(\tilde{{\boldsymbol{\theta }}}(s))[\dot{\tilde{{\boldsymbol{\theta }}}}(s)\dot{\tilde{{\boldsymbol{\theta }}}}(s)]+{{\boldsymbol{h}}}_{2}(\tilde{{\boldsymbol{\theta }}}(s))[\dot{\tilde{{\boldsymbol{\theta }}}}{(s)}^{2}]+\lambda {\boldsymbol{B}}\dot{\tilde{{\boldsymbol{\theta }}}}(s)\},\\ \,\,\simeq \frac{1}{{D}^{2}}\tilde{{\boldsymbol{\tau }}}(s),\end{array}$$

where $\tilde{{\boldsymbol{\tau }}}(s)$ is a time-normalised torque not related to the movement duration. Expressions have been developed using the general notation (*N*-joint); the two-joint case for shoulder and elbow is described in detail in Supplementary Equation 1 and Supplementary Fig. S3.

### Step 2: Determining the relationship between desired torque and torque noise

The actual neural process of human movement is affected by biological noise. Therefore, in the movement execution stage, we assume the presence of a signal-dependent noise represented by Gaussian noise with a mean of zero and a standard deviation proportional to the control signal that additively acts on the control signal^20^. Here, we refer to the signal-dependent noise at the torque level as the torque noise, ${\tau }_{i}^{{\rm{noise}}}$, which is represented as follows:

7 $${\tau }_{i}^{{\rm{noise}}}(t)={k}_{i}|{\tau }_{i}(t)|{z}_{i}(t),$$

where the noise parameter *k*_*i*_ indicates the degree to which the noise depends on the desired torque and *z*_*i*_(*t*) is a pseudo-random variable describing the standard normal distribution. Time-normalisation of Eq. (7) gives

8 $${\tau }_{i}^{{\rm{noise}}}(Ds)=\frac{1}{{D}^{2}}{k}_{i}|{\tilde{\tau }}_{i}(s)|{z}_{i}(s).$$

### Step 3: Determining the relationship between torque noise and hand endpoint error

The forward model is used to express a motor process in which endpoint error occurs as a result of the execution of a movement with noise-added torque. For instance, the forward model for a case of *x*-coordinate movement in a horizontal plane, ${{\boldsymbol{f}}}^{{\rm{FM}}}\in {{\mathbb{R}}}^{2\times 1}$, can be used to obtain the state variable of the hand, including position and velocity, ${\boldsymbol{X}}(t)={[x(t),\dot{x}(t)]}^{{\rm{T}}}\in {{\mathbb{R}}}^{2\times 1}$ from the joint torque ${\boldsymbol{\tau }}(t)={[{\tau }_{1}(t),{\tau }_{2}(t),\cdots ,{\tau }_{N}]}^{{\rm{T}}}\in {{\mathbb{R}}}^{N\times 1}$ as follows:

9 $$\frac{{\rm{d}}}{{\rm{d}}t}{\boldsymbol{X}}(t)={{\boldsymbol{f}}}^{{\rm{FM}}}({\boldsymbol{X}}(t),{\boldsymbol{\tau }}(t)).$$

Applying time integration from the beginning to the end of the movement (0 ≤ *t* ≤ *D*) to Eq. (9) and taking the difference between the equations with and without noise yields (see Supplementary Equation 2 for details):

10 $$\Delta {\boldsymbol{X}}(D)\simeq {\int }_{0}^{D}\frac{\partial {{\boldsymbol{f}}}^{{\rm{FM}}}({\boldsymbol{X}}(t),{\boldsymbol{\tau }}(t))}{\partial {\boldsymbol{\tau }}{(t)}^{{\rm{T}}}}\cdot {{\boldsymbol{\tau }}}^{{\rm{noise}}}(t){\rm{d}}t.$$

The partial derivative of the forward model ***f*** ^FM^ with respect to the torque ***τ*** is then approximated as follows (see Supplementary Equation 3 for details):

11 $$\frac{\partial {{\boldsymbol{f}}}^{{\rm{FM}}}({\boldsymbol{X}}(t),{\boldsymbol{\tau }}(t))}{\partial {\boldsymbol{\tau }}{(t)}^{{\rm{T}}}}\simeq [\begin{array}{c}{{\boldsymbol{J}}}^{x}({\boldsymbol{\theta }}(t)){{\boldsymbol{B}}}^{-1}\\ {{\boldsymbol{J}}}^{x}({\boldsymbol{\theta }}(t)){\boldsymbol{M}}{({\boldsymbol{\theta }}(t))}^{-1}\end{array}],$$

where ${{\boldsymbol{J}}}^{x}({\boldsymbol{\theta }}(t))$ is a Jacobian matrix from the joint space to the *x*-coordinate space. From Eq. (10), the positional endpoint error of hand state variable can be expressed in terms of the torque noise as follows:

12 $$\Delta x(D)\simeq \mathop{\sum }\limits_{i=1}^{N}\,{\int }_{0}^{D}{({{\boldsymbol{J}}}^{x}({\boldsymbol{\theta }}(t)){{\boldsymbol{B}}}^{-1})}_{i}{\tau }_{i}^{{\rm{noise}}}(t){\rm{d}}t,$$

where ${(\cdot )}_{i}$ is the vector in the *i*-th column (*i*-th joint) in the matrix shown in parentheses. By time-normalising Eq. (12) and substituting Eq. (8), the following relation is obtained:

13 $$\Delta x(D)\simeq \frac{1}{D}\mathop{\sum }\limits_{i=1}^{N}\,{k}_{i}{\alpha }_{i}^{x},\,{\alpha }_{i}^{x}={\int }_{0}^{1}{({{\boldsymbol{J}}}^{x}(\tilde{{\boldsymbol{\theta }}}(s)){{\boldsymbol{B}}}^{-1})}_{i}|{\tilde{\tau }}_{i}(s)|{z}_{i}(s){\rm{d}}s.$$

Similar derivations to those in Eqs. (9–13) can be applied to the *y*-coordinate:

14 $$\Delta y(D)\simeq \frac{1}{D}\mathop{\sum }\limits_{i=1}^{N}\,{k}_{i}{\alpha }_{i}^{y},\,{\alpha }_{i}^{y}={\int }_{0}^{1}{({{\boldsymbol{J}}}^{y}(\tilde{{\boldsymbol{\theta }}}(s)){{\boldsymbol{B}}}^{-1})}_{i}|{\tilde{\tau }}_{i}(s)|{z}_{i}(s){\rm{d}}s.$$

where ${\alpha }_{i}^{x}$ and ${\alpha }_{i}^{y}$ are constants in terms of *D* and are the values calculated by the dynamics parameters of the arm.

The model endpoint error *W* ^model^ in the horizontal plane is then expressed as the Euclidean distance between Δ*x*(*D*) and Δ*y*(*D*):

15 $$\begin{array}{rcl}{W}^{{\rm{model}}} & \equiv  & \sqrt{\Delta x{(D)}^{2}+\Delta y{(D)}^{2}},\\  & = & \frac{\gamma }{D},\,\gamma =\sqrt{{(\mathop{\sum }\limits_{i=1}^{N}{k}_{i}{\alpha }_{i}^{x})}^{2}+{(\mathop{\sum }\limits_{i=1}^{N}{k}_{i}{\alpha }_{i}^{y})}^{2}},\end{array}$$

where *γ* is calculated using the ${\alpha }_{i}^{x}$, ${\alpha }_{i}^{y}$, and *k*_*i*_. *k*_*i*_ is a signal-dependent noise parameter for each joint. ${\alpha }_{i}^{x}$ and ${\alpha }_{i}^{y}$ are calculated using the following dynamics parameters:

- Kinematic parameters: the time-normalised joint angular position ${\tilde{\theta }}_{i}(s)$, velocity ${\dot{\tilde{\theta }}}_{i}(s)$, and acceleration ${\ddot{\tilde{\theta }}}_{i}(s)$ for each joint.
- Physical parameters: the *i*-th link length *L*_*i*_, distance from the joint to the centre of mass *S*_*i*_, mass *m*_*i*_, moment of inertia *I*_*i*_, and joint viscosity coefficient *B*_*ij*_.

### Physical meanings of Fitts’ law coefficients

As a further consideration, we consider the physical meanings of the Fitts’ law coefficients, *a* and *b*. As these coefficients represent the relationship between speed and accuracy, they have been used as an index of human motor performance. Therefore, examining the coefficients of Fitts’ law is an important step to gaining a deeper understanding of human motor performance.

Solving Eq. (15) for *D* gives

16 $$D=\frac{\gamma }{{W}^{{\rm{model}}}}.$$

To bring this equation into the Fitts’ law form, we first consider a first-order Taylor expansion of ln (1 + *x*) around *x* = 0; $\mathrm{ln}\,(1+x)\simeq x$, for −1 < *x* ≤ 1. By setting $x=\frac{c}{{W}^{{\rm{model}}}}-1$, we obtain

17 $$\mathrm{ln}(\frac{c}{{W}^{{\rm{model}}}})\simeq \frac{c}{{W}^{{\rm{model}}}}-1,\,{\rm{for}}\,0 < \frac{c}{{W}^{{\rm{model}}}}\le 2.$$

where *c* is a constant. From the change-of-base formula, the following linear approximation is obtained:

18 $$\begin{array}{ll}\frac{1}{{W}^{{\rm{model}}}}\simeq {p}_{1}+{p}_{2}\,{\log }_{2}(\frac{1}{{W}^{{\rm{model}}}}), & {\rm{for}}\,\frac{c}{2}\le {W}^{{\rm{model}}},\\ {p}_{1}=\frac{1}{c}(1+\,\mathrm{ln}\,c), & \\ {p}_{2}=\frac{1}{c\,{\log }_{2}e}, & {\rm{where}}\,e\,{\rm{is}}\,{\rm{Napier}}\mbox{'}{\rm{s}}\,{\rm{constant}}\,(e\simeq 2.71828\cdots ).\end{array}$$

If *c* = 0.0060, the range of *W* ^model^ is 0.003 m ≤ *W* ^model^ and *p*_1_ and *p*_2_ are determined as −686.0 and 115.5, respectively. Once the value of *c* is determined, *p*_1_ and *p*_2_ are irrelevant to the subject or task. Finally, the approximation of the model to Fitts’ law is represented by

19 $$D={a}^{{\rm{model}}}+{b}^{{\rm{model}}}\,{\log }_{2}(\frac{1}{{W}^{{\rm{model}}}}),$$

20 $${a}^{{\rm{model}}}={p}_{1}\gamma ,$$

21 $${b}^{{\rm{model}}}={p}_{2}\gamma .$$

The above equations show that *γ* is equally involved in both *a*^model^ and *b*^model^, which correspond to the intercept and slope of Fitts’ law, respectively. Therefore, the slope and intercept of Fitts’ law can be expressed by arm dynamics and signal-dependent noise parameters. Previous studies have observed that the slope and intercept of Fitts’ law are related to, for example, arm components (such as fingers, wrists, forearms and full arm) and arm physical parameters (such as length, mass, the moment of inertia)^11–17^ and, therefore, such empirical findings might be revealed theoretically by further study.

Because *c* is always positive, *p*_2_ is always positive; thus, increasing *γ* increases the Fitts’ law slope, and vice versa. We therefore consider the case in which *γ* increases. From Eq. (15), *γ* increases when $|{\sum }_{i=1}^{N}\,{k}_{i}{\alpha }_{i}^{x}|$ or $|{\sum }_{i=1}^{N}\,{k}_{i}{\alpha }_{i}^{y}|$ increases. Both terms contain the signal-dependent noise parameter *k*_*i*_, which is always positive, so that *γ* increases as *k*_*i*_ increases; that is, the slope of Fitts’ law increases as the noise level increases. Focusing on the ${\alpha }_{i}^{x}$ and ${\alpha }_{i}^{y}$ calculated by the arm dynamics parameters, *γ* can also be reduced by reducing ${\alpha }_{i}^{x}$ and ${\alpha }_{i}^{y}$, even if *k*_*i*_ has a large value. There are two ways to reduce ${\alpha }_{i}^{x}$ and ${\alpha }_{i}^{y}$—reduce the absolute value of the time-normalised torque or reduce the product of the Jacobian matrix and the inverse viscosity matrix—but it is difficult to intuitively understand how to reduce these owing to the nonlinearity of the dynamics. To understand these factors, it is preferable to rely on numerical calculations such as sensitivity analysis.

## Results

The relationship between movement duration and hand endpoint error could be modelled using *W* ^model^ = *γ*/*D*. We first investigated how the degree of hand endpoint error calculated by the model could represent the hand endpoint error observed in actual movement. We then examined how close the model function is to Fitts’ law when it was applied to the Fitts’ law axis, log_2_ (1/*W*) − *D*.

The behavioural experiment involved reaching the target at four points on the horizontal plane (front, back, left and right, as viewed from the starting point) within a given movement duration range. Eleven subjects participated, and the distance between the starting point and target point was fixed at 15 cm. The shoulder, elbow and hand positions were measured using a three-dimensional optical position measurement digitizer. The experiment was continued until a total of 320 successful trials (i.e., 80 trials in each direction) had been conducted (see Methods for details of the experiment).

### How well the degree of model endpoint error can represent the actual endpoint error

**Figure 2:**
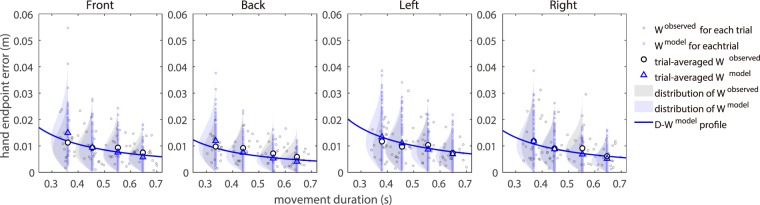
Comparison between observed and model endpoint errors for a representative subject (A). Tiny grey circles show observed endpoint error for each observed movement duration. Open circles show averaged values of observed endpoint errors at the mean observed movement duration. Tiny light blue crosses show the model endpoint errors at the mean observed movement duration using different pseudorandom numbers. When calculating this, the average of 20 measured trajectories in each movement duration was used as the time-normalised trajectory. Blue triangles show the trial-averaged values of the model endpoint error at the mean observed movement duration. The blue line shows *D* − *W* function of the model, which is obtained by using the mean value of *γ* over the entire movement duration (i.e., 0.30–0.70 s). In calculating this, the average of 80 measured trajectories over the entire movement duration was used as the time-normalised trajectory. Histograms hatched in translucent grey and blue are the distributions after fitting the observed and model endpoint error data with a Rayleigh distribution. As the *x*- and *y*-axis endpoint errors follow a Gaussian distribution, their Euclidean distance (i.e., *W*) follows a Rayleigh distribution.

We examined how the degree of endpoint error calculated by the model can explain the endpoint error observed in actual movement. Figure 2 shows a comparison between the observed and model endpoint errors in a representative subject (see Supplementary Figs. S4 to S13 for other subjects). Table 1 lists the root mean square errors (RMSEs) between the model and observed endpoint error for each subject. The model represented the trial-averaged observed endpoint error well, with a small average error of 0.0043 m. Subjects A, E and K had small RMSEs in all directions (average RMSE less than 0.0030 m), while C, F and H had high RMSEs in all directions (average RMSE greater than 0.0060 m). Overall, the model endpoint error closely matched the observed endpoint errors of actual movement. The observed and model endpoint errors for single-trial are not in one-to-one correspondence (the observed endpoint errors were measured for 20 trials per movement duration condition, while the model endpoint errors were generated over 100 trials using different pseudorandom numbers). The distributions of endpoint errors in the observed and model results were roughly consistent.

**Table 1:** RMSE between model endpoint error and observed endpoint error for each subject.

RMSE (m)												
Subject	A	B	C	D	E	F	G	H	I	J	K	Mean ± SD*
Front	0.0022	0.0028	0.0043	0.0035	0.0028	0.0087	0.0031	0.0040	0.0022	0.0043	0.0038	0.0038 ± 0.0017
Back	0.0019	0.0032	0.0050	0.0031	0.0018	0.0130	0.0028	0.0051	0.0031	0.0019	0.0028	0.0040 ± 0.0031
Left	0.0013	0.0037	0.0076	0.0068	0.0029	0.0073	0.0043	0.0071	0.0055	0.0032	0.0016	0.0047 ± 0.0022
Right	0.0012	0.0026	0.0089	0.0057	0.0043	0.0074	0.0033	0.0075	0.0067	0.0037	0.0027	0.0049 ± 0.0023
Mean	0.0017	0.0031	0.0064	0.0048	0.0029	0.0091	0.0034	0.0060	0.0044	0.0033	0.0027	0.0043 ± 0.0020

*SD: standard deviation across subjects.

Among the four directions, leftward endpoint variability was large while backward endpoint variability was small; overall, the model captured these direction-dependent features of hand endpoint variability. The direction dependency of the differences could be attributed to variations in the viscosity coefficient *B*_*ij*_ and time-normalised trajectories $\tilde{\theta }(s)$, velocities $\dot{\tilde{\theta }}(s)$, and accelerations $\ddot{\tilde{\theta }}(s)$, as all other physical parameters (*L*_*i*_, *S*_*i*_, *m*_*i*_, *I*_*i*_) and signal-dependent noise parameters (*k*_*i*_) were the same in all directions.

### How close the model function is to Fitts’ law

**Figure 3:**
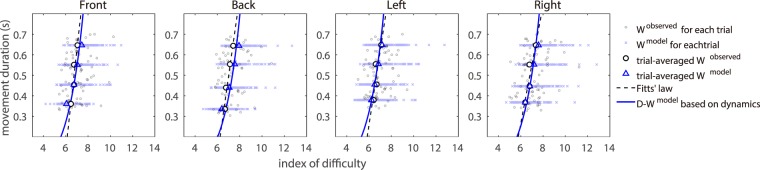
Comparison between Fitts’ law and the model in *I*_*d*_ − *D* axis for a representative subject (A). The tiny grey and open circles, light blue crosses and blue triangles represent the same factors shown in Fig. 2. The black dashed and blue solid lines indicate the Fitts’ law results (regression line for the trial-averaged *W* ^observed^) and the model line along the *I*_*d*_ − *D* axis, respectively.

We investigated how close the model, *D* = *γ*/*W* ^model^, is to Fitts’ law, *D* = *a*′ + *b*log_2_ (1/*W*) along the log_2_ (1/*W*) − *D* axis. Figure 3 shows a simple conversion of the *D* − *W* axes shown in Fig. 2 to log_2_ (1/*W*) − *D* axes. Fitts’ law (black-dashed line) is indicated by a straight line, while the model line (blue solid line) is somewhat concave along the axis of Fitts’ law, log_2_ (1/*W*) − *D*. This concavity has been observed in many previous studies^3,4,11,14,25^. The functions are quite close to each other. Similar results were obtained for several other subjects (see Supplementary Figs. S4 to S13 for other subjects’ results).

A comparison of the parameters describing Fitts’ law (*a*′ and *b*) with the corresponding parameters for the model (*a*^model^ and *b*^model^) revealed average slope values over the subjects and directions of *b* = 0.340 and *b*^model^ = 0.431, respectively, and average intercept values over the subjects and directions of *a*′ = −1.752 and *a*^model^ = −2.558, respectively. Thus, in terms of subject-averaged values, both *a*^model^ and *b*^model^ were close to the Fitts’ law values of *a*′ and *b*, respectively. These results indicate that Fitts’ law can be explained by the *W* − *D* relation derived based on human arm dynamics.

## Discussion

The relationship between movement duration *D* and hand endpoint error *W*—the factors used in Fitts’ law—were modelled based on human arm dynamics and found to follow an inversely proportional form: *W* = *γ*/*D* (where *γ* is a coefficient based on arm dynamics and signal-dependent noise parameters). The model was found to nearly coincided with Fitts’ law when plotted on the axes log_2_ (1/*W*) and *D*. Furthermore, it was theoretically shown that the slope and intercept of Fitts’ law include both arm dynamics and noise parameters based on transformation of the model into the Fitts’ law form. In the field of Human-Computer Interaction and Ergonomics, the slope and intercept of Fitts’ law have been used as an index of human motor performance because they represent the relationship between speed and accuracy. Our research results are significant in providing a first step to determining a specific relationship between the motor performance and arm dynamics.

Harris and Wolpert^20^ and Tanaka *et al*.^21^ showed that the SAT profile drawn by Fitts’ law can be reproduced using their respectively proposed optimisation models. These were important results showing that Fitts’ law for one-dimensional reaching movements can be reproduced based on single-joint arm dynamics. However, the multi-joint arm dynamics used in the more general arm movements performed by humans on a daily basis can cause nonlinear interference torques such as Coriolis and centrifugal forces between the upper arm and the forearm. As such the nonlinear interference torques do not occur under single-joint arm dynamics, it is important to investigate their effect on the SAT profile based on multi-joint arm dynamics. Our model, which is based on multi-joint arm dynamics, considers both Coriolis and centrifugal forces and therefore should be able to reproduce actual human arm movement more accurately than a model that does not consider them. To verify this, we compared applications of the model with and without Coriolis and centrifugal forces. An initial comparison using the data measured in the experiment described in Methods revealed an insignificant amount of difference between the two cases (Supplementary Fig. S14). This occurred because the inertial and viscous forces dominated the Coriolis and centrifugal forces during this task and because the Coriolis and centrifugal forces in the shoulder joint cancelled each other in the Front and Back directions (Supplementary Fig. S15). We then conducted an experiment with different starting and target postures to analyse the effects of moving rapidly over a longer distance. Supplementary Fig. S16 shows the results of the comparison for each subject. For five of the six subjects, the trial-averaged values of the actual endpoint error were better represented using the model with Coriolis and centrifugal forces, which also produced results closer the actual distributions of endpoint errors. These results demonstrate the importance of considering Coriolis and centrifugal forces in modelling very rapid reaching movements over long distances and highlight one of the advantages of our model, namely, that it considers the nonlinearity of arm movement.

Our model is based on the following assumptions and approximations: (1) feedforward control is assumed; (2) it is assumed that movement of the upper arm and forearm in the horizontal plane can be modelled by the dynamics of a two-link planar manipulator; (3) trajectory invariance in which the trajectory does not change within a certain range of movement duration is assumed; (4) the viscous force $\frac{1}{D}B\dot{\tilde{\theta }}(s)$ is approximated by $\frac{1}{{D}^{2}}\lambda B\dot{\tilde{\theta }}(s)$ within a certain range of movement duration (Eq. (6); (5) the presence of signal-dependent noise is assumed (Eq. (7)); (6) a first-order Taylor approximation of the forward model around ${\tau }_{i}^{{\rm{n}}oise}\mathrm{=0}$ is applied (Supplementary Equation 2); (7) it is assumed that the effect of the partial derivatives of Coriolis and centrifugal forces are negligible (Supplementary Equation 3); and (8) a linear approximation is applied to formulate Fitts’ law (Eq. (18)). These assumptions and approximations give several limitations to the model. For example, the effects of feedback cannot be considered because of assumption (1). Moreover, (3), (4), (6), (7) and (8) lead to modelling errors. It is also necessary to discuss the validity of the trajectory invariance assumption in (3). Whether or not time-normalisation is a valid must be confirmed in terms of how much the actually measured trajectory changes over the duration of movement. Supplementary Fig. S1 shows the measured hand paths of all subjects when moving a distance of 15 cm from 0.30 to 0.70 s. Although this provides a qualitative description, it can be considered to indicate that there is no problem with the changes even if trajectory invariance is assumed. This assumption is approximately valid to within a certain range of movement speeds that are neither too slow nor too fast. It should be noted that the assumption does not hold for very rapid or slow movement and, therefore, that finding a method that can be formulated without using time-normalisation should be undertaken as a future task. Another issue to be addressed in the future is the lack of a sophisticated understanding of motor impedance sources such as viscosity and stiffness. In this study, we did not consider joint stiffness because doing so would complicate the model. Given the numerous studies showing that stiffness is related to the SAT^26–28^, it will be important to incorporate stiffness into future versions of the model. Despite the limitations noted above, the error between the model and the experimental results was small, and we can conclude that the model can successfully explain the endpoint error occurring in actual movement.

The use of signal-dependent noise made it possible to represent the SATs—a breakthrough in the study of motor control. This breakthrough should be supplemented by further findings obtained, for example, by considering the constant and temporal noises in the model, as van Beers *et al*.^22^ did; and considering how to estimate the noise parameter *k* using data other than hand endpoint error (e.g., a method that can estimate using EMG signals). We are also interested in studying human motor control strategies that achieve high spatial accuracy even with very rapid movement. It is speculated that nonlinear interference forces such as Coriolis and centrifugal forces are effectively used in such strategies, but this needs to be studied in detail.

Finally, in this study we investigated only arm-reaching movement. It will be possible to investigate other types of movement, such as eye and feet movement, have not, but changing the approach, for example, from arm to foot dynamics. The relationships derived in this paper can therefore be generally applied to any type of movement, which will be an important approach in future research.

## Methods

We conducted behavioural experiments to examine to what degree the model endpoint error can represent the actual endpoint error and how similar the model is to Fitts’ law when it is applied to the Fitts’ law axis.

### Subjects

Eleven healthy young adults (11 males, age range 21–24 y) participated. All subjects were right-handed according to the Edinburgh handedness test score (score range 64.7–100%). Informed consent agreements were obtained from all subjects, and the study was conducted according to the guidelines of the Declaration of Helsinki and approved by the Ethics Committee of Nagaoka University of Technology.

### Apparatus

**Figure 4:**
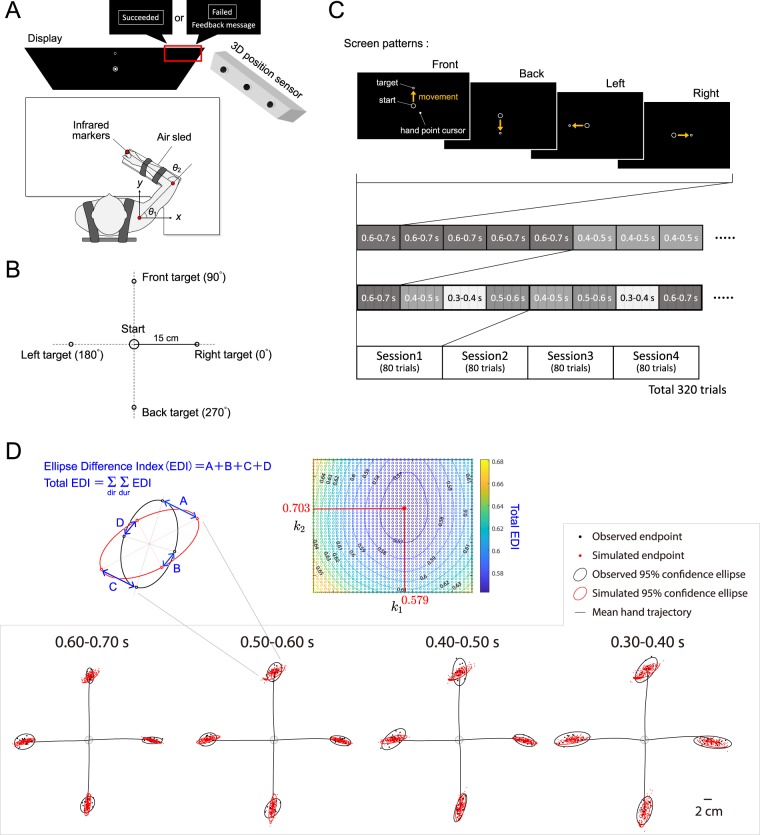
Experimental design. (**A**) Top view of experimental setup, showing definitions of joint angles *θ*_1_ and *θ*_2_ and *x*- and *y*-coordinates. (**B**) Display positions of all four targets with respect to central circle. (**C**) Examples of screen patterns of starting and target positions and experimental protocols. (**D**) Process for estimating signal-dependent noise parameters *k*_1_ and *k*_2_. To evaluate the degree of coincidence between pairs of ellipses, we defined the Ellipse Difference Index (EDI), which calculates the distances between four corresponding points (**A**–**D**) on the respective ellipses. The sum of the EDI over all conditions of movement duration and direction was set as the total EDI, with the values of *k*_1_ and *k*_2_ at the minimum total EDI selected as the signal-dependent noises.

The experimental setup is illustrated in Fig. 4A. The subjects sat on the chair located in front of the table and display. Each subject placed his right arm on the air-sled on the table to reduce the friction between their arm and the table, with the hand position set to match the tip of the handle of the air-sled. The air-sled and forearm were then fixed with stretch tape and the chair height was adjusted so that the table and subject’s arm were oriented in a parallel manner. Each subject’s shoulder was restrained by seat belts, restricting movement of the arm to the horizontal plane. The chair was then moved toward the table until the subject’s chest just touched the table. Infrared markers were affixed to the shoulder and elbow joints and the hand, and the three marker positions were measured at a sampling frequency of 500 Hz using a three-dimensional optical position measurement digitiser (Optotrak Certus, Northern Digital Inc., Waterloo, Canada). The measured current hand positions were projected onto a display (PDP-504P, Pioneer, Tokyo, Japan) installed in front of the subject. A starting point (a circle with a radius of 10 mm) and a target (a circle with a radius of 2 mm) were also shown on the screen, and the subjects performed the experimental task while watching the display.

### Task

Experimental tasks used in the study of SAT primarily fall into two categories: spatially constrained movement tasks, and temporally constrained movement tasks (details are given in^4^).

In this study, we used a temporally constrained movement task based on our assumption of pre-programmed and rapid movements. The task involved setting a target movement duration *D* and movement distance *A* and then measuring the hand endpoint error or variability *W* of the corresponding movement. The subjects were required to reach the target as accurately as possible within the instructed range of movement duration.

Specifically, as shown in Fig. 4A, the task involved using the shoulder and elbow joints to engage in arm reaching movements in the horizontal plane toward the target, a circle with a radius of 2 mm, located 15 cm away from a 10-mm starting circle displayed in the centre of the screen. The initial shoulder position was defined as the origin of a coordinate system in which the positive *x*- and *y*-axes were to the right and in front of the subject, respectively. The starting position was defined as a shoulder angle (*θ*_1_) of 45° and an elbow angle (*θ*_2_) of 100°, and therefore the starting position relative to the right shoulder differed by subject: on average, it was −0.072 m toward the *x*-axis and 0.390 m toward the *y*-axis with respect to the initial shoulder position.

The targets were located at four directions—front (90°), back (270°), left (180°), and right (0°)—relative to the starting position (Fig. 4B). Movement was measured over the target movement duration intervals 0.30–0.40, 0.40–0.50, 0.50–0.60 and 0.60–0.70 s, respectively. As the distance from the starting point to the target was fixed, the movement speed was controlled. After the end of each trial, it was determined whether the measured movement duration was within the set duration range and whether the movement was executed ballistically. If both conditions were satisfied, the trial was regarded as a success; if neither was satisfied, it was regarded as a failure. Corresponding feedback was displayed to the subject on the upper right of the screen as follows: *failure* (*too fast*), *failure* (*too slow*), or *success* (Fig. 4A).

Figure 4C shows examples of the screen for each target pattern; the four target patterns were presented pseudorandomly. In the example shown, the pattern started from the front and, if the trial was a success, it continued backward, leftward and then rightward. The conditions of target movement duration were also applied pseudorandomly; in the example, the first interval was 0.60–0.70 s and was repeated five times until success was achieved in all four directions. If success was achieved over 4 directions × 5 rounds = 20 trials, the next movement duration was applied. If success was achieved at all movement duration conditions, the session was concluded. In all, four sessions were performed for each subject. As it was necessary for each subject to succeed at 80 trials per session, the experiment was continued until a total of 320 successful trials had been conducted.

Each subject participated in a preparatory experiment involving the same conditions as the main experiment but with half the number of sessions, with the goal of familiarising the subjects with the experimental setup and imparting a sense of target movement duration. The data measured in the preparatory experiment were not used in the subsequent analysis; in any event, no apparent learning effects were observed in the main experiments as a result of the preparatory sessions.

### Data analysis

#### Observed endpoint error and movement duration

Our methods for pre-processing the positional data and determining the observed endpoint error and movement duration were carried out as followed. The acquired positional data were low-pass filtered using a third-order, zero phase-lag Butterworth filter with a cut-off frequency of 10 Hz. The start and end of each movement were determined based on the tangential velocity, which was calculated using numerical differentiation. The start of a movement was defined as the point following the start cue at which the tangential velocity first exceeded the threshold velocity, defined as 5% of the peak value of the tangential velocity. The end of a movement was defined as the point prior to the end cue at which the tangential velocity fell below the threshold. The time from movement start to end was then defined as the observed movement duration, *D*. The required conditions of a successful trial were therefore (1) that the observed movement duration fell within the target range and (2) that there was only one peak in the tangential velocity between the start and end of the movement. The successful trials were then analysed to determine the corresponding Euclidean distances from the centre of the target to the endpoint, which was defined as the observed endpoint error, *W* ^observed^.

#### Model endpoint error

To obtain the model endpoint error, *W* ^model^, the following model parameters were used: the kinematic parameters such as the time-normalised desired angular position, velocity, and acceleration for each joint (${\tilde{\theta }}_{i}(s)$, ${\dot{\tilde{\theta }}}_{i}(s)$, and ${\ddot{\tilde{\theta }}}_{i}(s)$), the physical parameters for each link or joint (*L*_*i*_, *S*_*i*_, *m*_*i*_, *I*_*i*_, and *B*_*ij*_), and the noise parameter for each joint (*k*_*i*_). We describe below how we estimated them.

Desired trajectories. We used the joint angular trajectories averaged over trials as the theoretically desired trajectory. The joint angular trajectory was first calculated using the measured positional data and subject’s arm length by inverse kinematics and resampled temporally at the mean movement duration for each trial. The trial-averaged trajectories were then calculated for each subject and direction (for each target movement duration was also calculated as well). The trial-averaged joint angular velocities and acceleration were also calculated and time-normalised in terms of *D* to $\dot{\tilde{\theta }}(s)$ and $\ddot{\tilde{\theta }}(s)$, respectively.

Physical parameters. The upper arm and forearm lengths, *L*_1_ and *L*_2_, respectively, were obtained from the distances between the markers on the shoulder, elbow, and hand, which were measured using the position digitiser before starting the experiment. The distance from the joint to the centre of mass, *S*_*i*_, mass, *m*_*i*_, and moment of inertia around the joint, *I*_*i*_, were estimated based on their respective proportional relationships to arm length^19,24,29^. An adjusted forearm mass, *m*_2_, was obtained by adding the 0.740-kg mass of the air-sled and stretch tapes to the forearm mass estimated using the above method. The viscosity coefficient, *B*_*ij*_, was estimated using the method applied in^19^ based on the approximate relationship between joint torque and viscosity coefficient during static force control, as measured by^30^. The resulting physical parameters of the subject’s arm are listed in Table 2. Note that the viscosity coefficients shown in the table were obtained using the trial-averaged joint angular trajectory for each direction. For further details on how to calculate these physical parameters, refer to Supplementary Equation 4.

**Table 2:** Physical parameters of the arm and signal-dependent noise parameters.

Subject	A	B	C	D	E	F	G	H	I	J	K	Mean ± SD*
*L*_1_ (m)	0.302	0.270	0.264	0.261	0.266	0.308	0.274	0.278	0.296	0.303	0.282	0.282 ± 0.017
*L*_2_ (m)	0.325	0.315	0.335	0.335	0.312	0.350	0.316	0.319	0.345	0.360	0.332	0.331 ± 0.016
*S*_1_ (m)	0.114	0.101	0.098	0.097	0.099	0.116	0.102	0.104	0.111	0.114	0.106	0.106 ± 0.007
*S*_2_ (m)	0.159	0.155	0.164	0.164	0.153	0.170	0.155	0.156	0.168	0.175	0.162	0.162 ± 0.007
*m*_1_ (kg)	1.506	1.332	1.299	1.283	1.310	1.538	1.354	1.375	1.473	1.511	1.397	1.398 ± 0.093
*m*_2_ (kg)	1.794	1.764	1.824	1.824	1.755	1.868	1.767	1.776	1.853	1.898	1.815	1.813 ± 0.046
*I*_1_ (kg · m^2^)	0.030	0.021	0.019	0.019	0.020	0.032	0.022	0.023	0.028	0.030	0.024	0.024 ± 0.005
*I*_2_ (kg · m^2^)	0.040	0.037	0.043	0.043	0.036	0.049	0.037	0.038	0.047	0.053	0.042	0.042 ± 0.006
**Front**												
*B*_11_ (kg · m^2^/s)	0.698	0.682	0.685	0.684	0.684	0.708	0.681	0.686	0.693	0.698	0.694	0.690 ± 0.009
*B*_12_ (kg · m^2^/s)	0.180	0.181	0.181	0.181	0.180	0.183	0.181	0.182	0.182	0.182	0.179	0.181 ± 0.001
*B*_21_ (kg · m^2^/s)	0.180	0.181	0.181	0.181	0.180	0.183	0.181	0.182	0.182	0.182	0.179	0.181 ± 0.001
*B*_22_ (kg · m^2^/s)	0.784	0.789	0.789	0.791	0.787	0.799	0.790	0.794	0.795	0.794	0.779	0.790 ± 0.006
**Back**												
*B*_11_ (kg · m^2^/s)	0.680	0.668	0.672	0.672	0.671	0.688	0.668	0.667	0.671	0.681	0.679	0.674 ± 0.007
*B*_12_ (kg · m^2^/s)	0.186	0.185	0.186	0.187	0.185	0.190	0.185	0.185	0.187	0.188	0.186	0.186 ± 0.002
*B*_21_ (kg · m^2^/s)	0.186	0.185	0.186	0.187	0.185	0.190	0.185	0.185	0.187	0.188	0.186	0.186 ± 0.002
*B*_22_ (kg · m^2^/s)	0.813	0.808	0.816	0.818	0.811	0.833	0.808	0.810	0.821	0.823	0.813	0.816 ± 0.007
**Left**												
*B*_11_ (kg · m^2^/s)	0.747	0.729	0.733	0.723	0.723	0.766	0.734	0.733	0.753	0.759	0.742	0.740 ± 0.015
*B*_12_ (kg · m^2^/s)	0.180	0.180	0.181	0.180	0.180	0.181	0.179	0.180	0.181	0.181	0.182	0.180 ± 0.001
*B*_21_ (kg · m^2^/s)	0.180	0.180	0.181	0.180	0.180	0.181	0.179	0.180	0.181	0.181	0.182	0.180 ± 0.001
*B*_22_ (kg · m^2^/s)	0.784	0.784	0.789	0.787	0.784	0.789	0.779	0.783	0.791	0.792	0.796	0.787 ± 0.005
**Right**												
*B*_11_ (kg · m^2^/s)	0.722	0.709	0.704	0.704	0.703	0.737	0.709	0.706	0.720	0.727	0.720	0.715 ± 0.011
*B*_12_ (kg · m^2^/s)	0.181	0.180	0.181	0.181	0.181	0.181	0.180	0.180	0.181	0.182	0.182	0.181 ± 0.001
*B*_21_ (kg · m^2^/s)	0.181	0.180	0.181	0.181	0.181	0.181	0.180	0.180	0.181	0.182	0.182	0.181 ± 0.001
*B*_22_ (kg · m^2^/s)	0.788	0.787	0.792	0.790	0.787	0.790	0.783	0.786	0.790	0.795	0.797	0.790 ± 0.004
*k*_1_	0.559	0.537	0.540	0.171	0.606	0.679	0.667	0.300	0.235	0.601	0.571	0.497 ± 0.177
*k*_2_	0.667	0.656	0.929	0.737	0.654	0.907	0.732	0.890	0.728	0.750	0.715	0.760 ± 0.101

*SD: standard deviation across subjects.

Noise parameters. The parameter determining the magnitude of signal-dependent noise, *k*_*i*_, was estimated using the following simulation procedure:

1. The joint torques were calculated using the mean trajectories and the physical parameters of the arm.
2. Signal-dependent noise was generated artificially using an arbitrary value of *k*_*i*_ and added to the joint torque. The calculation applied Eq. (7) with *z*_*i*_(*t*) set as a random variable with a standard normal distribution.
3. The endpoint errors were obtained by transforming the noise-added torque from the joint to the task space using the forward dynamics and kinematics models.
4. Step 1–3 were repeated 100 times for each pairing of *k*_1_ and *k*_2_ to obtain 100 simulated endpoints. We then used a grid search algorithm to find the value of *k*_*i*_ corresponding to the smallest difference between the confidence ellipses of the simulated and observed endpoints in terms of the Ellipse Difference Index (EDI), the sum of distances between four corresponding points on two ellipses (see Fig. 4D). The total EDI was defined as the summed value of the EDI over all movement duration and directions.

The noise parameters of each subject obtained using this method are listed at the bottom of Table 2.

## Supplementary information

Supplementary Information

## Data Availability

The datasets used and/or analysed during the current study are available from the corresponding author on reasonable request.
