# Supplementary material for: Explanation of Fitts’ law in Reaching Movement based on Human Arm Dynamics
Source: Sci Rep. 2019 Dec 24;9:19804. doi: 10.1038/s41598-019-56016-7 (PMC6930222; doi:10.1038/s41598-019-56016-7)
Supplement: Supplementary file 1 — Supplementary Information [file 41598_2019_56016_MOESM1_ESM.pdf]

# Supplementary information

## Explanation of Fitts' law in Reaching Movement based on Human Arm Dynamics

Misaki Takeda[1,*], Takanori Sato[1,2], Hisashi Saito[1], Hiroshi Iwasaki[1], Isao Nambu[1], Yasuhiro Wada[1]

[1] Graduate School of Engineering, Nagaoka University of Technology, Nagaoka, Niigata, 940-2188, Japan
[2] National Institute of Technology, Akita College, Akita, Akita, 011-8511, Japan

[*] To whom correspondence should be addressed: Misaki Takeda: Graduate School of Engineering, Nagaoka University of Technology, Nagaoka, Niigata, 940-2188, Japan; tmisaki@stn.nagaokaut.ac.jp.

## Supplementary Equations 1: Derivation in the case of two-joint arm dynamics

To express movement of the upper arm and forearm, i.e., the shoulder and elbow joint ($N = 2$), the arm dynamic model shown in Supplementary Fig. S3 is assumed. Equation (3) is concretely written as follows[18]:

$$\begin{aligned}\tau_1(t) = &\left(I_1 + I_2 + 2m_2L_1S_2\cos\theta_2(t) + m_2{L_1}^2\right)\ddot{\theta}_1(t) + \left(I_2 + m_2L_1S_2\cos\theta_2(t)\right)\ddot{\theta}_2(t)\\ &- m_2L_1S_2\left(2\dot{\theta}_1(t) + \dot{\theta}_2(t)\right)\dot{\theta}_2(t)\sin\theta_2(t) + B_{11}\dot{\theta}_1(t) + B_{12}\dot{\theta}_2(t),\end{aligned} \tag{22}$$

$$\tau_2(t) = \left(I_2 + m_2L_1S_2\cos\theta_2(t)\right)\ddot{\theta}_1(t) + I_2\ddot{\theta}_2(t) + m_2L_1S_2\dot{\theta}_1(t)^2\sin\theta_2(t) + B_{21}\dot{\theta}_1(t) + B_{22}\dot{\theta}_2(t), \tag{23}$$

where the subscript $i$ represents the respective links, with $i = 1,2$ corresponding to the upper arm and forearm, respectively, and $L_i$, $S_i$, $m_i$, $I_i$, and $B_{ij}$ representing length, distance from the joint to the centre of mass, mass, moment of inertia around the joint for each link, and viscosity coefficient, respectively. Because the path taken by the hand during human arm movement is nearly invariant within a specific range of movement duration[23], the following time-normalisation can be assumed:

$$t = Ds,\ \ 0 \le t \le D \quad (0 \le s \le 1), \quad \theta_i(t) = \theta_i(Ds) = \tilde{\theta}_i(s). \tag{4}$$

It follows that

$$\dot{\theta}_i(t) = \frac{\mathrm{d}}{\mathrm{d}t}\theta_i(t) = \frac{\mathrm{d}s}{\mathrm{d}t}\frac{\mathrm{d}}{\mathrm{d}s}\tilde{\theta}_i(s) = \frac{1}{D}\dot{\tilde{\theta}}_i(s), \tag{24}$$

$$\ddot{\theta}_i(t) = \frac{\mathrm{d}}{\mathrm{d}t}\dot{\theta}_i(t) = \frac{1}{D}\frac{\mathrm{d}s}{\mathrm{d}t}\frac{\mathrm{d}}{\mathrm{d}s}\dot{\tilde{\theta}}_i(s) = \frac{1}{D^2}\ddot{\tilde{\theta}}_i(s). \tag{25}$$

Equations (22) and (23) can then be expressed in terms of $D$ as follows:

$$\tau_i(t) = \tau_i(Ds) = \frac{1}{D^2}\left(\tilde{\tau}_i^{\text{inertial}} + \tilde{\tau}_i^{\text{Coriolis}} + \tilde{\tau}_i^{\text{centri}}\right) + \frac{1}{D}\tilde{\tau}_i^{\text{viscous}}, \tag{26}$$

where

$$\tilde{\tau}_1^{\text{inertial}} = \left(I_1 + I_2 + 2m_2L_1S_2\cos\tilde{\theta}_2(s) + m_2{L_1}^2\right)\ddot{\tilde{\theta}}_1(s) + \left(I_2 + m_2L_1S_2\cos\tilde{\theta}_2(s)\right)\ddot{\tilde{\theta}}_2(s), \tag{27}$$

$$\tilde{\tau}_2^{\text{inertial}} = \left(I_2 + m_2L_1S_2\cos\tilde{\theta}_2(s)\right)\ddot{\tilde{\theta}}_1(s) + I_2\ddot{\tilde{\theta}}_2(s), \tag{28}$$

$$\tilde{\tau}_1^{\text{Coriolis}} = -2m_2L_1S_2\dot{\tilde{\theta}}_1(s)\dot{\tilde{\theta}}_2(s)\sin\tilde{\theta}_2(s), \qquad \tilde{\tau}_2^{\text{Coriolis}} = 0, \tag{29}$$

$$\tilde{\tau}_1^{\text{centri}} = -m_2L_1S_2\dot{\tilde{\theta}}_2(s)^2\sin\tilde{\theta}_2(s), \qquad \tilde{\tau}_2^{\text{centri}} = m_2L_1S_2\dot{\tilde{\theta}}_1(s)^2\sin\tilde{\theta}_2(s), \tag{30}$$

$$\tilde{\tau}_1^{\text{viscous}} = B_{11}\dot{\tilde{\theta}}_1(s) + B_{12}\dot{\tilde{\theta}}_2(s), \qquad \tilde{\tau}_2^{\text{viscous}} = B_{21}\dot{\tilde{\theta}}_1(s) + B_{22}\dot{\tilde{\theta}}_2(s). \tag{31}$$

As shown in Equation (26), the torque of each joint ($\tau_i$) is expressed as the sum of the reciprocal of the square of movement duration ($1/D^2$) multiplied by the sum of the time-normalised inertial force ($\tilde{\tau}_i^{\text{inertial}}$), Coriolis force ($\tilde{\tau}_i^{\text{Coriolis}}$), and centrifugal force ($\tilde{\tau}_i^{\text{centri}}$) and the product of the time-normalised viscous force ($\tilde{\tau}_i^{\text{viscous}}$) and inverse movement duration ($1/D$).

## Supplementary Equations 2: Derivation from Equation (9) to Equation (10)

The forward model $\boldsymbol{f}^{\mathrm{FM}}$ for obtaining the state of the hand including position and velocity $\boldsymbol{X}(t) = [x(t), \dot{x}(t)]^{\mathrm{T}}$ from the joint torque $\boldsymbol{\tau}(t) = [\tau_1(t), \tau_2(t), \cdots, \tau_N]^{\mathrm{T}}$ is given by

$$\frac{\mathrm{d}}{\mathrm{d}t}\boldsymbol{X}(t) = \boldsymbol{f}^{\mathrm{FM}}\left(\boldsymbol{X}(t), \boldsymbol{\tau}(t)\right). \tag{9}$$

Time integration is then performed on Equation (9) from the start to the end of movement ($0 \leq t \leq D$) to obtain

$$\boldsymbol{X}(D) - \boldsymbol{X}(0) = \int_0^D \boldsymbol{f}^{\mathrm{FM}}\left(\boldsymbol{X}(t), \boldsymbol{\tau}(t)\right)\mathrm{d}t. \tag{32}$$

The corresponding hand state variable of the endpoint error $\Delta\boldsymbol{X}(D) \in \mathbb{R}^{2\times 1}$ is then expressed using the noise $\boldsymbol{\tau}^{\mathrm{noise}}(t) \in \mathbb{R}^{N\times 1}$ as follows:

$$\Delta\boldsymbol{X}(D) = \int_0^D \boldsymbol{f}^{\mathrm{FM}}\left(\boldsymbol{X}(t), \boldsymbol{\tau}(t) + \boldsymbol{\tau}^{\mathrm{noise}}(t)\right)\mathrm{d}t - \int_0^D \boldsymbol{f}^{\mathrm{FM}}\left(\boldsymbol{X}(t), \boldsymbol{\tau}(t)\right)\mathrm{d}t. \tag{33}$$

Based on the first-order Taylor approximation of $\boldsymbol{f}^{\mathrm{FM}}$ around $\boldsymbol{\tau}^{\mathrm{noise}} = \mathbf{0}$

$$\boldsymbol{f}^{\mathrm{FM}}\left(\boldsymbol{X}(t), \boldsymbol{\tau}(t) + \boldsymbol{\tau}^{\mathrm{noise}}(t)\right) \simeq \boldsymbol{f}^{\mathrm{FM}}\left(\boldsymbol{X}(t), \boldsymbol{\tau}(t)\right) + \frac{\partial \boldsymbol{f}^{\mathrm{FM}}\left(\boldsymbol{X}(t), \boldsymbol{\tau}(t)\right)}{\partial \boldsymbol{\tau}(t)^{\mathrm{T}}} \cdot \boldsymbol{\tau}^{\mathrm{noise}}(t), \tag{34}$$

Equation (33) becomes

$$\Delta\boldsymbol{X}(D) \simeq \int_0^D \frac{\partial \boldsymbol{f}^{\mathrm{FM}}\left(\boldsymbol{X}(t), \boldsymbol{\tau}(t)\right)}{\partial \boldsymbol{\tau}(t)^{\mathrm{T}}} \cdot \boldsymbol{\tau}^{\mathrm{noise}}(t)\mathrm{d}t. \tag{10}$$

## Supplementary Equations 3: Partial derivative of forward model with respect to joint torque

The partial derivative of the forward model $\boldsymbol{f}^{\mathrm{FM}}$ in $x$-coordinate space with respect to the joint torque $\boldsymbol{\tau}$ is expressed as

$$\begin{aligned}
\frac{\partial \boldsymbol{f}^{\mathrm{FM}}\left(\boldsymbol{X}(t), \boldsymbol{\tau}(t)\right)}{\partial \boldsymbol{\tau}(t)^{\mathrm{T}}} &= \begin{bmatrix} \dfrac{\partial \dot{x}(t)}{\partial \boldsymbol{\tau}(t)^{\mathrm{T}}} \\ \dfrac{\partial \ddot{x}(t)}{\partial \boldsymbol{\tau}(t)^{\mathrm{T}}} \end{bmatrix} = \begin{bmatrix} \dfrac{\partial \dot{x}(t)}{\partial \dot{\boldsymbol{\theta}}(t)^{\mathrm{T}}} \dfrac{\partial \dot{\boldsymbol{\theta}}(t)}{\partial \boldsymbol{\tau}(t)^{\mathrm{T}}} \\ \dfrac{\partial \ddot{x}(t)}{\partial \ddot{\boldsymbol{\theta}}(t)^{\mathrm{T}}} \dfrac{\partial \ddot{\boldsymbol{\theta}}(t)}{\partial \boldsymbol{\tau}(t)^{\mathrm{T}}} \end{bmatrix} = \begin{bmatrix} \boldsymbol{J}^{x}\left(\boldsymbol{\theta}(t)\right) \dfrac{\partial \dot{\boldsymbol{\theta}}(t)}{\partial \boldsymbol{\tau}(t)^{\mathrm{T}}} \\ \boldsymbol{J}^{x}\left(\boldsymbol{\theta}(t)\right) \dfrac{\partial \ddot{\boldsymbol{\theta}}(t)}{\partial \boldsymbol{\tau}(t)^{\mathrm{T}}} \end{bmatrix} = \begin{bmatrix} \boldsymbol{J}^{x}\left(\boldsymbol{\theta}(t)\right) \left( \dfrac{\partial \boldsymbol{\tau}(t)}{\partial \dot{\boldsymbol{\theta}}(t)^{\mathrm{T}}} \right)^{-1} \\ \boldsymbol{J}^{x}\left(\boldsymbol{\theta}(t)\right) \left( \dfrac{\partial \boldsymbol{\tau}(t)}{\partial \ddot{\boldsymbol{\theta}}(t)^{\mathrm{T}}} \right)^{-1} \end{bmatrix} \\
&= \begin{bmatrix} \boldsymbol{J}^{x}\left(\boldsymbol{\theta}(t)\right) \boldsymbol{G}\left(\boldsymbol{\theta}(t), \dot{\boldsymbol{\theta}}(t)\right)^{-1} \\ \boldsymbol{J}^{x}\left(\boldsymbol{\theta}(t)\right) \boldsymbol{M}\left(\boldsymbol{\theta}(t)\right)^{-1} \end{bmatrix},
\end{aligned} \tag{35}$$

where $\boldsymbol{J}^{x}\left(\boldsymbol{\theta}(t)\right) \in \mathbb{R}^{1 \times N}$ is a Jacobian matrix from the joint space to the $x$-coordinate space. For a two-link arm with shoulder and elbow joints ($N = 2$), it is given as

$$\boldsymbol{J}^{x}\left(\boldsymbol{\theta}(t)\right) = \begin{bmatrix} -L_1 \sin \theta_1(t) - L_2 \sin\left(\theta_1(t) + \theta_2(t)\right) & -L_2 \sin\left(\theta_1(t) + \theta_2(t)\right) \end{bmatrix}, \tag{36}$$

where $\boldsymbol{M}\left(\boldsymbol{\theta}(t)\right)^{-1} \in \mathbb{R}^{N \times N}$ is the inverse inertia matrix from Equation (3) and $\boldsymbol{G}\left(\boldsymbol{\theta}(t), \dot{\boldsymbol{\theta}}(t)\right)^{-1} \in \mathbb{R}^{N \times N}$ is a matrix that transforms from the joint torque space to a joint angular velocity space, which is expressed from equations (22) and (23) as follows:

$$\begin{aligned}
\boldsymbol{G}\left(\boldsymbol{\theta}(t), \dot{\boldsymbol{\theta}}(t)\right)^{-1} &= \left( \frac{\partial \boldsymbol{h}_1\left(\boldsymbol{\theta}(t)\right) \left[\dot{\boldsymbol{\theta}}(t) \dot{\boldsymbol{\theta}}(t)\right]}{\partial \dot{\boldsymbol{\theta}}(t)^{\mathrm{T}}} + \frac{\partial \boldsymbol{h}_2\left(\boldsymbol{\theta}(t)\right) \left[\dot{\boldsymbol{\theta}}(t)^2\right]}{\partial \dot{\boldsymbol{\theta}}(t)^{\mathrm{T}}} + \frac{\partial \boldsymbol{B} \dot{\boldsymbol{\theta}}(t)}{\partial \dot{\boldsymbol{\theta}}(t)^{\mathrm{T}}} \right)^{-1}, \\
&= \begin{bmatrix} -2 m_2 L_1 S_2 \sin \theta_2(t) \dot{\theta}_2(t) + B_{11} & -2 m_2 L_1 S_2 \sin \theta_2(t) \left(\dot{\theta}_1(t) + \dot{\theta}_2(t)\right) + B_{12} \\ 2 m_2 L_1 S_2 \sin \theta_2(t) \dot{\theta}_1(t) + B_{21} & B_{22} \end{bmatrix}^{-1}.
\end{aligned} \tag{37}$$

As it is assumed that the effect of the partial derivative of the Coriolis force $\frac{\partial \boldsymbol{h}_1(\boldsymbol{\theta}(t))\left[\dot{\boldsymbol{\theta}}(t)\dot{\boldsymbol{\theta}}(t)\right]}{\partial \dot{\boldsymbol{\theta}}(t)^{\mathrm{T}}}$ and the partial derivative of the centrifugal force $\frac{\partial \boldsymbol{h}_2(\boldsymbol{\theta}(t))\left[\dot{\boldsymbol{\theta}}(t)^2\right]}{\partial \dot{\boldsymbol{\theta}}(t)^{\mathrm{T}}}$ are small, Equation (37) becomes

$$\boldsymbol{G}\left(\boldsymbol{\theta}(t), \dot{\boldsymbol{\theta}}(t)\right)^{-1} \simeq \boldsymbol{B}^{-1} = \begin{bmatrix} B_{11} & B_{12} \\ B_{21} & B_{22} \end{bmatrix}^{-1}. \tag{38}$$

Thus, Equation (12) is obtained.

## Supplementary Equations 4: Determining the physical parameters of the arm

The distance from the joint to the centre of mass ($S_i$), mass ($m_i$), and moment of inertia around the joint ($I_i$) were estimated using the proportional relationship of these parameters to the arm length ($L_i$) based on the physical parameters of a specific subject[24] (see Supplementary Table S1). The respective parameters were calculated as follows:

$$S_i = S_i^{\text{base}} \frac{l_i}{L_i^{\text{base}}}, \quad m_i = m_i^{\text{base}} \frac{l_i}{L_i^{\text{base}}}, \quad I_i = I_i^{\text{base}} \left( \frac{l_i}{L_i^{\text{base}}} \right)^3, \tag{39}$$

where $l_1 = L_1 - 0.025$ and $l_2 = L_2 + 0.030$.

**Supplementary Table S1.** Baseline physical parameters

| Parameter | Link 1 | Link 2 |
|---|---|---|
| $L_i^{\text{base}}$ (m) | 0.2750 | 0.3570 |
| $S_i^{\text{base}}$ (m) | 0.1130 | 0.1600 |
| $m_i^{\text{base}}$ (kg) | 1.4950 | 1.0600 |
| $I_i^{\text{base}}$ ($\text{kg} \cdot \text{m}^2$) | 0.0294 | 0.0405 |

## Supplementary Figures

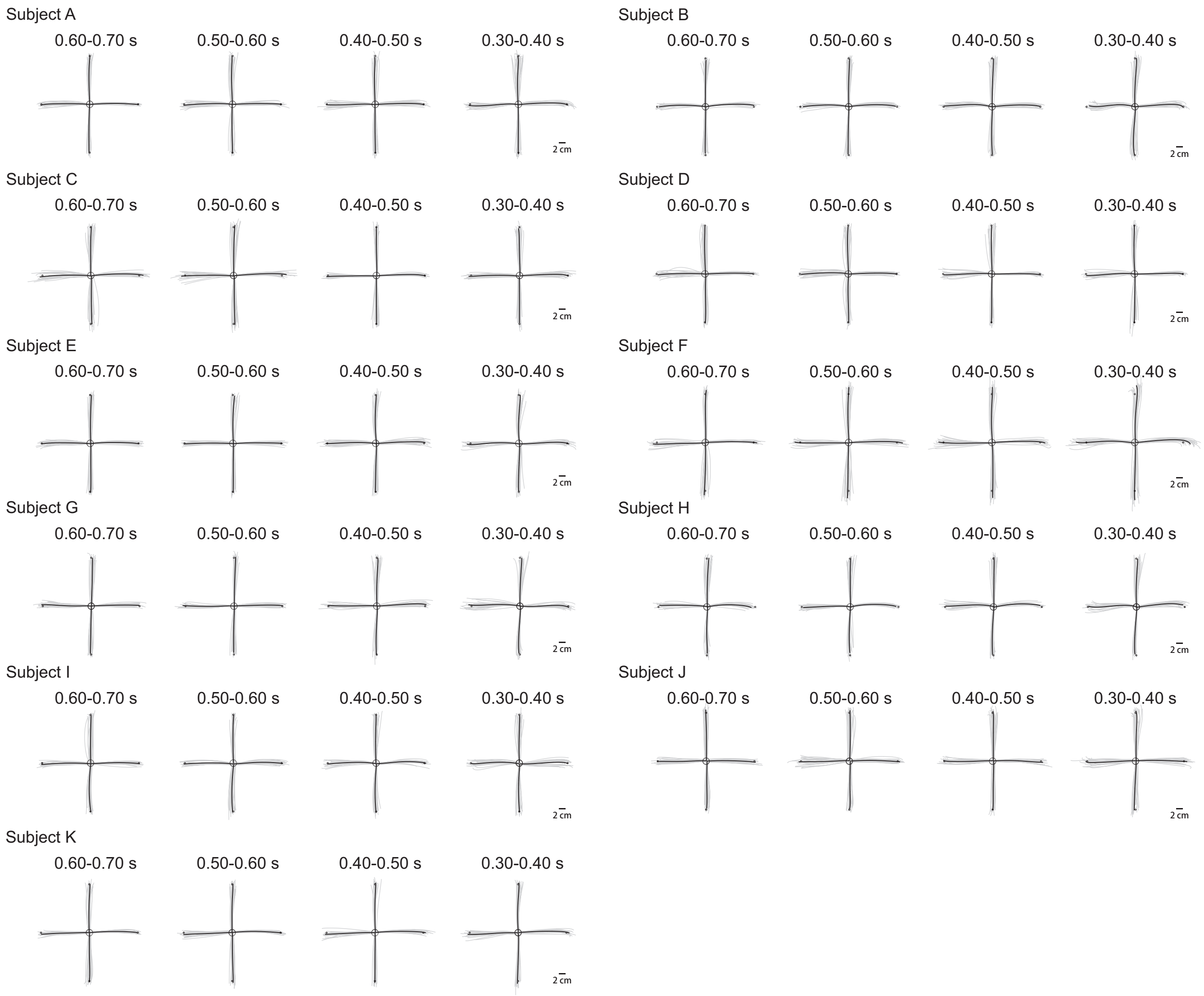


**Supplementary Figure S1.** Observed hand paths for each target movement duration for each subject.

## Supplementary Figures

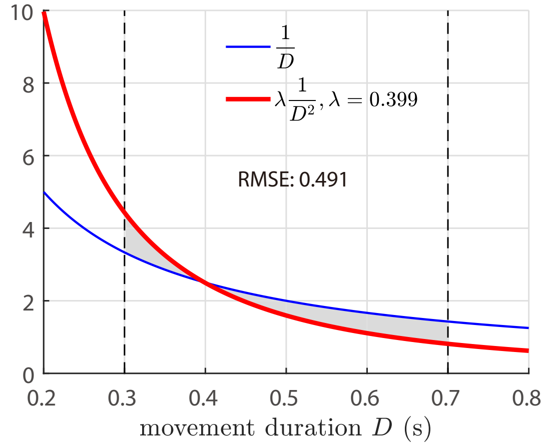


**Supplementary Figure S2.** Effect of approximation error in Equation (6), in which the approximation $\frac{1}{D} \simeq \lambda \frac{1}{D^2}$ is used. As the approximation range of movement duration $D$ is from 0.30 to 0.70 s, $\lambda = 0.399$ (the coefficient $\lambda$ is determined using the least squares method). The RMSE between $1/D$ and $0.399/D^2$ during this time range is 0.491. Thus, the proposed model is always affected by this approximation error.

## Supplementary Figures

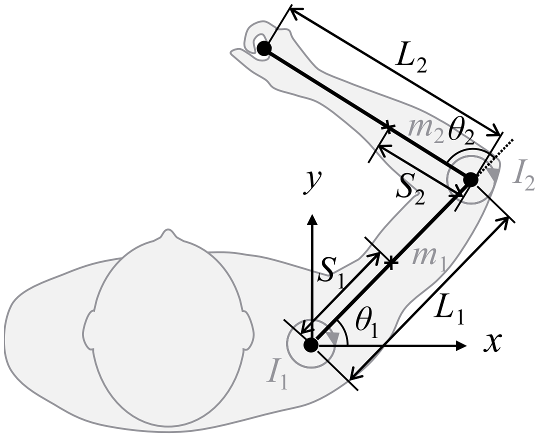


**Supplementary Figure S3.** Arm dynamics model with two-joints (shoulder and elbow).

## Supplementary Figures

## Comparison of observed and model endpoint errors for each subject

In the main text, the results for subject A were shown in Figs. 2 and 3 as representative for the test group. The results obtained for the remaining 10 subjects (B to K) are shown in Supplementary Figs. S4 to S13. Among these 10 subjects, B (except Left direction), D (except Right direction), E, F, G, I, J and K were observed to follow Fitts' law, while C and H did not. For B, C and H in particular, there was a tendency toward larger movement errors during slow movements, such as those from 0.50–0.60 s and from 0.60–0.70 s, than during rapid movements, such as from 0.30–0.40 s and from 0.40–0.50 s. A potential cause of this effect was that the subjects had to adapt to a unique experimental environment in the behavioural experiments in which air-sled were used to reduce the frictional resistance between the arm and table. Although we performed a preparatory experiment prior to the main experiment, some of the subjects may not have been able to produce sufficiently-learned movement data as a result of this environment.

For the subject group for which Fitts' law held (B, D, E, F, G, I, J and K), the model was able to estimate values close to the actual movement endpoint error. However, the results of Left and Right directions for G did not coincide, while F's overall model values were underestimates of the actual results. These discrepancies potentially arose from errors in the estimation of model parameters such as noise and dynamics parameters. Further consideration will be needed to determine a method for estimating model parameters more accurately.

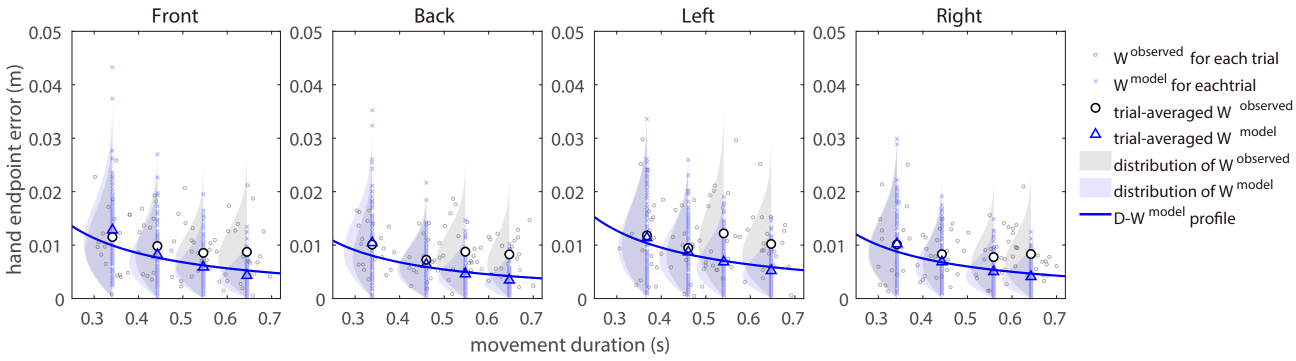


**(a)** *D–W* profile

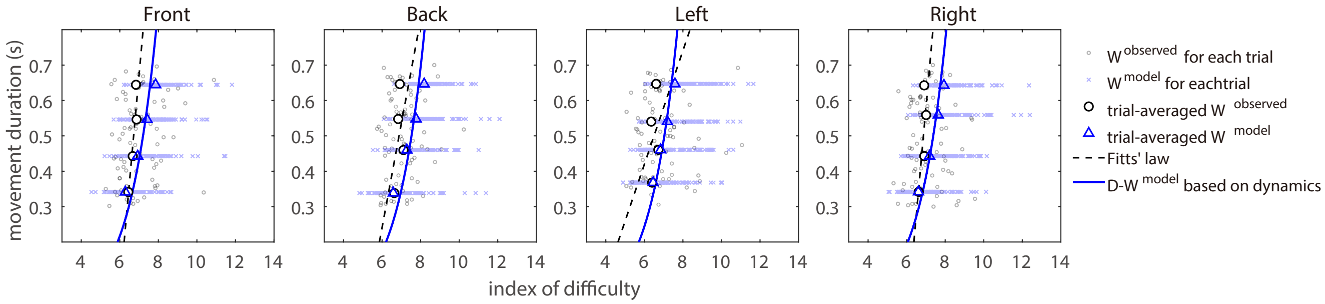


**(b)** $\log_2\left(\frac{1}{W}\right)$–$D$ profile (Fitts' law)

**Supplementary Figure S4.** Subject B

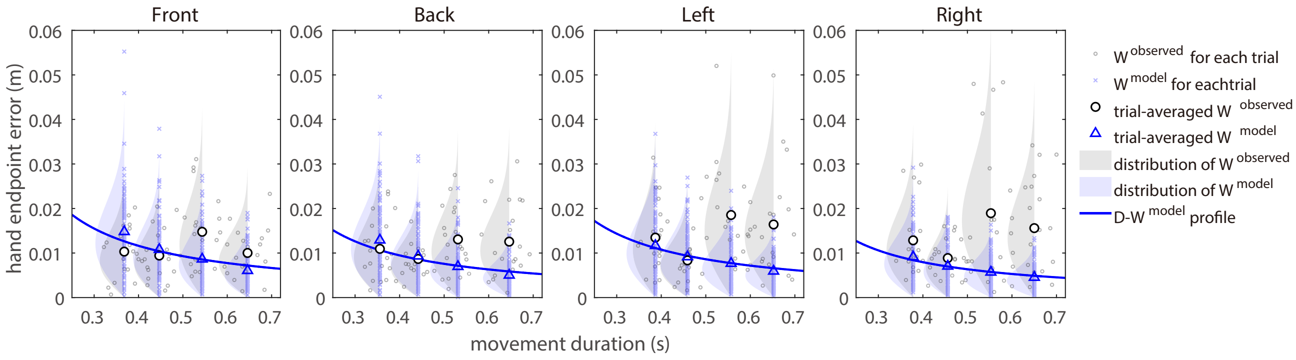


**(a)** *D–W* profile

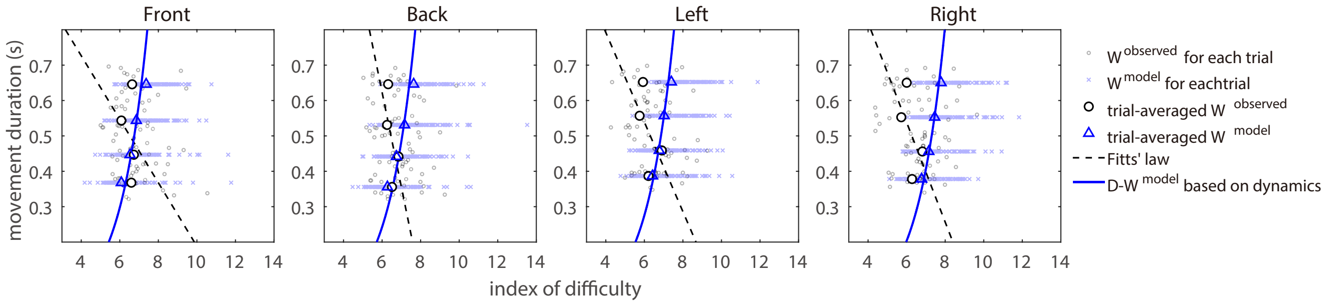


**(b)** $\log_2\left(\frac{1}{W}\right)$–$D$ profile (Fitts' law)

**Supplementary Figure S5.** Subject C

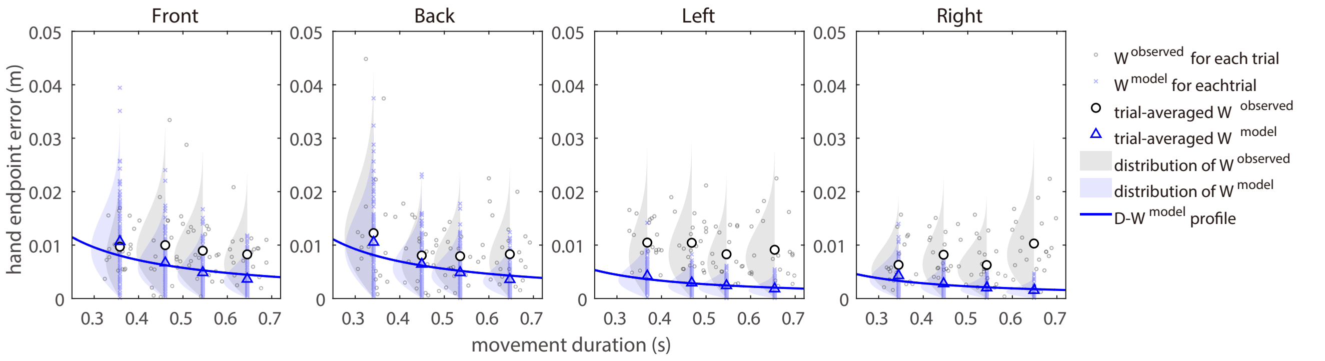


**(a)** $D$–$W$ profile

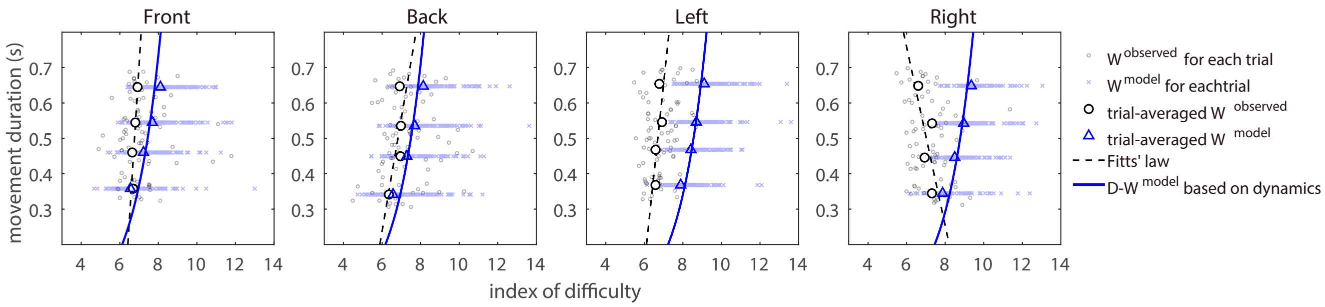


**(b)** $\log_2\left(\frac{1}{W}\right)$–$D$ profile (Fitts' law)

**Supplementary Figure S6.** Subject D

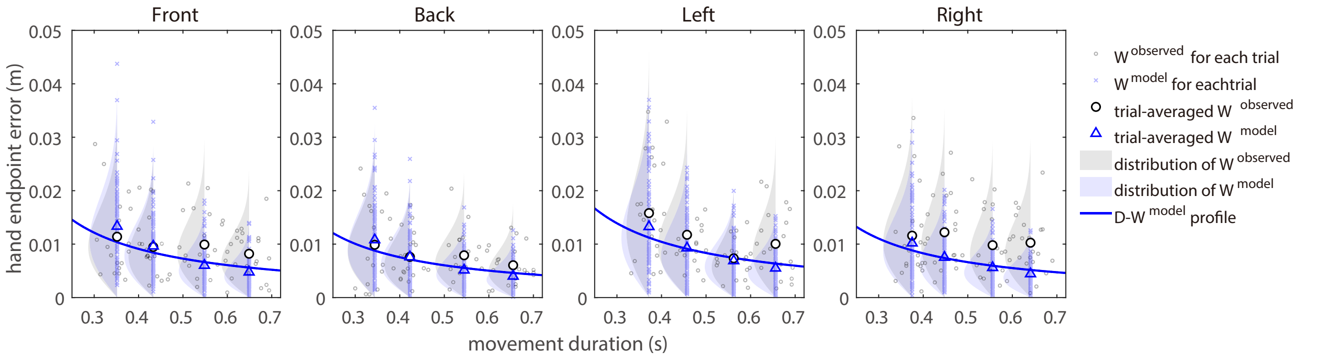


**(a)** $D$–$W$ profile

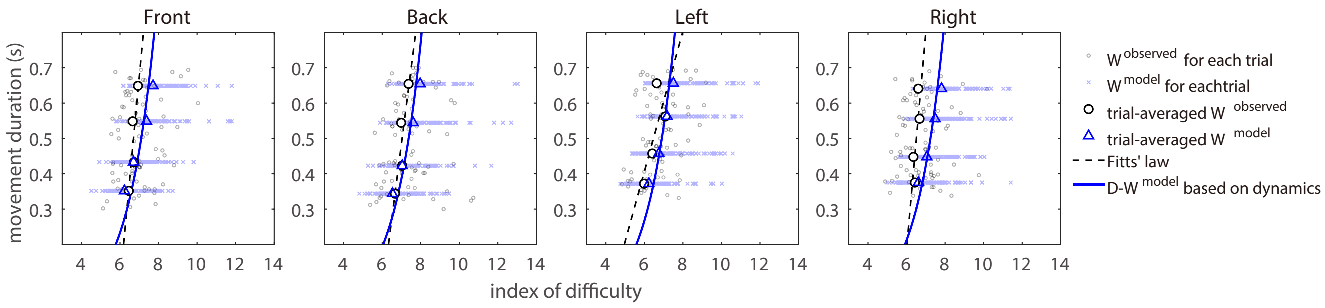


**(b)** $\log_2\left(\frac{1}{W}\right)$–$D$ profile (Fitts' law)

**Supplementary Figure S7.** Subject E

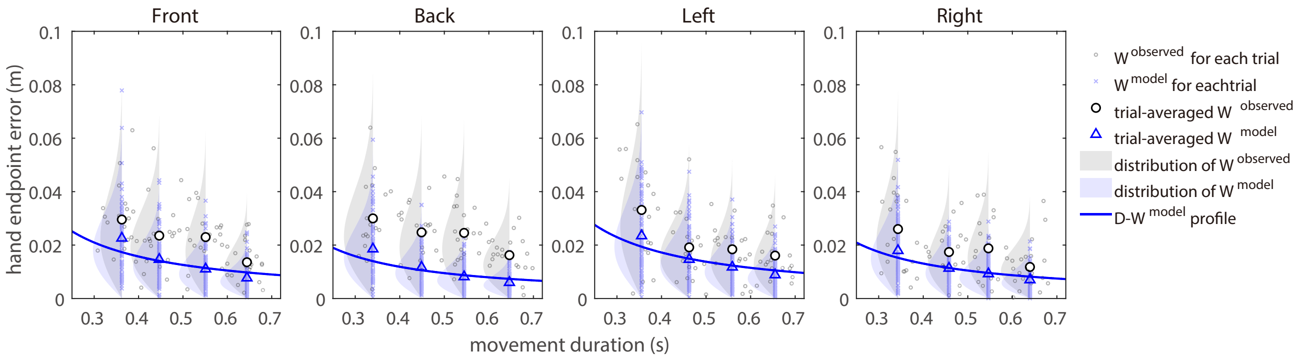


**(a)** *D–W* profile

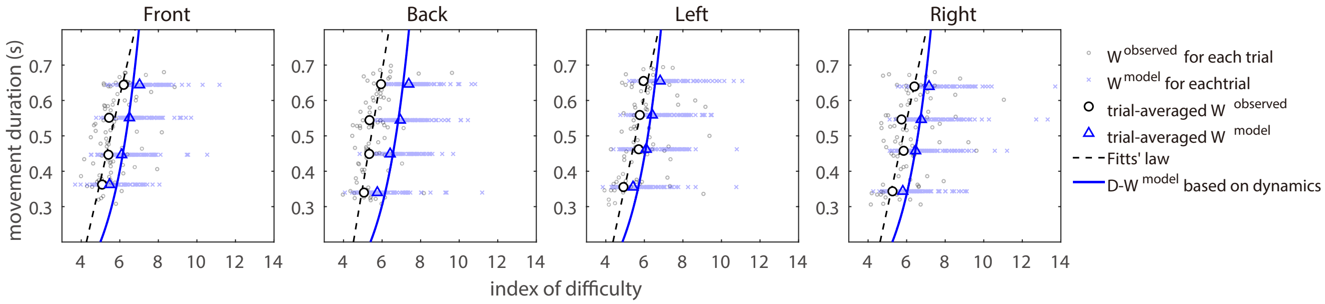


**(b)** $\log_2\left(\frac{1}{W}\right)$–$D$ profile (Fitts' law)

**Supplementary Figure S8.** Subject F

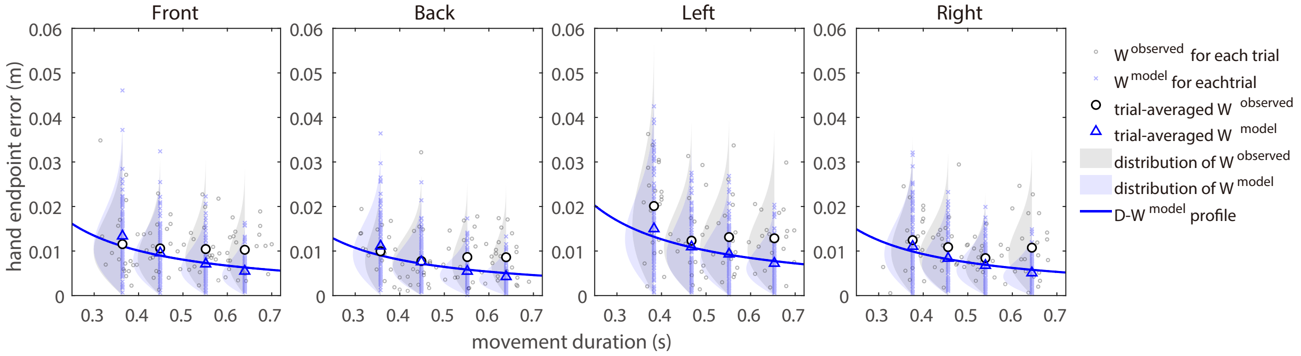


**(a)** *D–W* profile

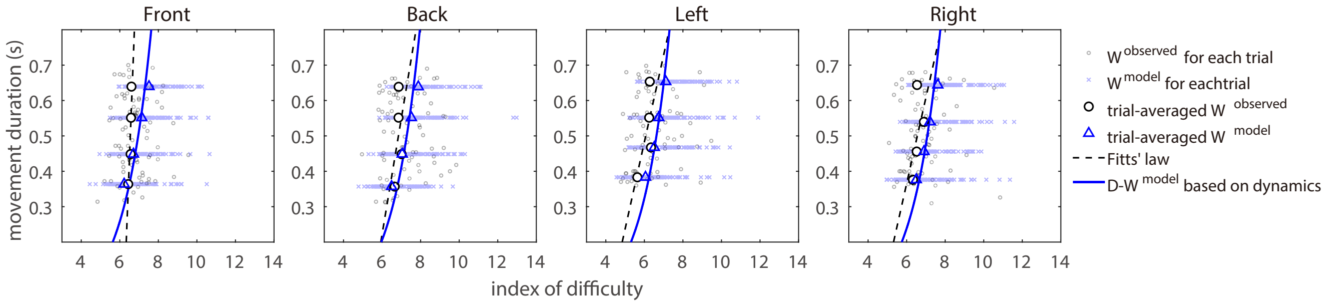


**(b)** $\log_2\left(\frac{1}{W}\right)$–$D$ profile (Fitts' law)

**Supplementary Figure S9.** Subject G

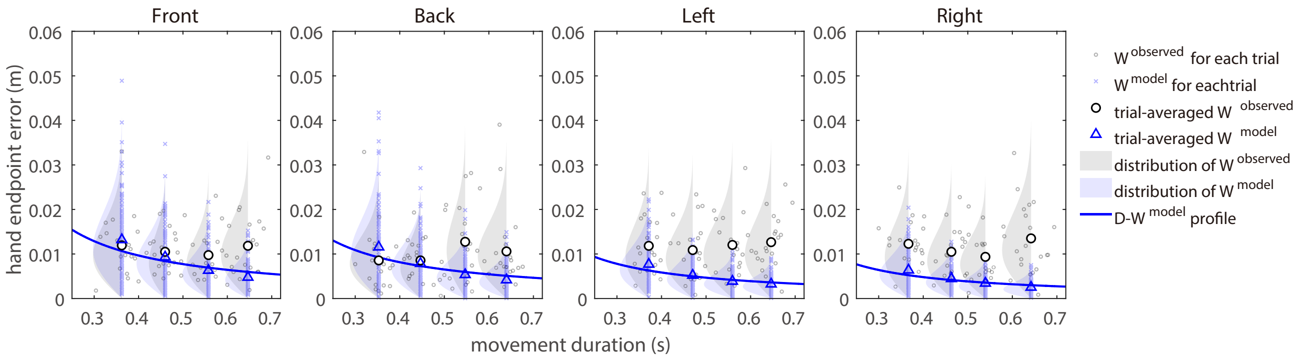


**(a)** $D$–$W$ profile

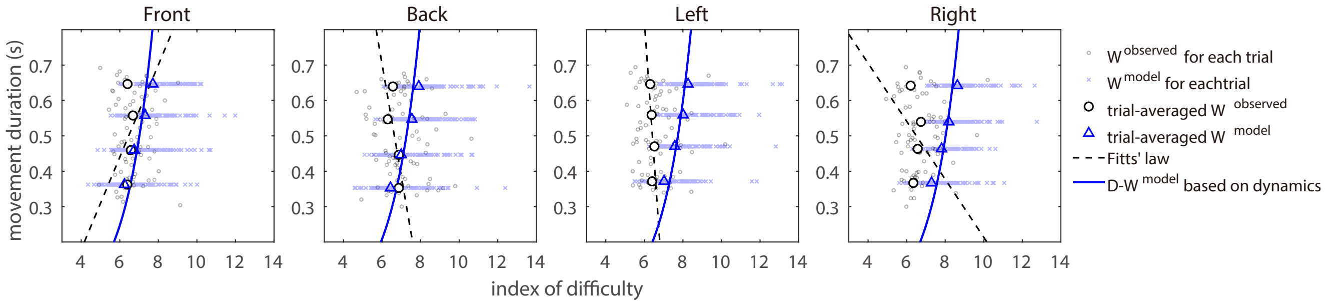


**(b)** $\log_2\left(\frac{1}{W}\right)$–$D$ profile (Fitts' law)

**Supplementary Figure S10.** Subject H

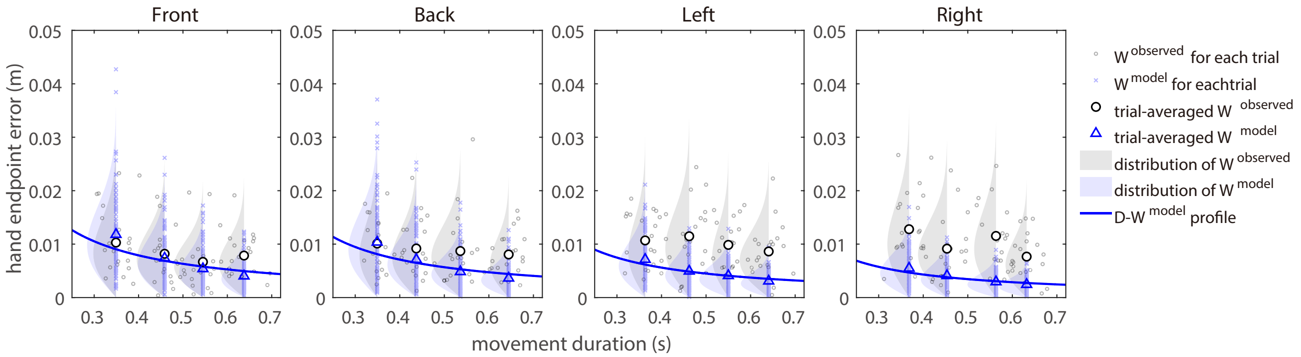


**(a)** $D$–$W$ profile

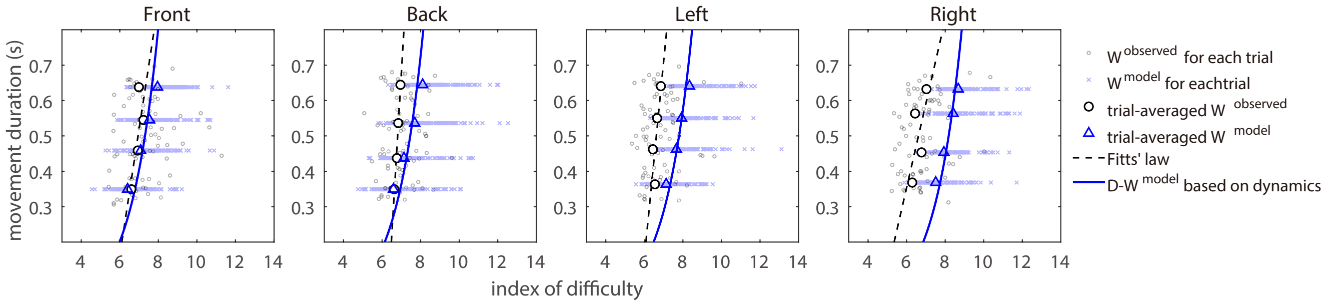


**(b)** $\log_2\left(\frac{1}{W}\right)$–$D$ profile (Fitts' law)

**Supplementary Figure S11.** Subject I

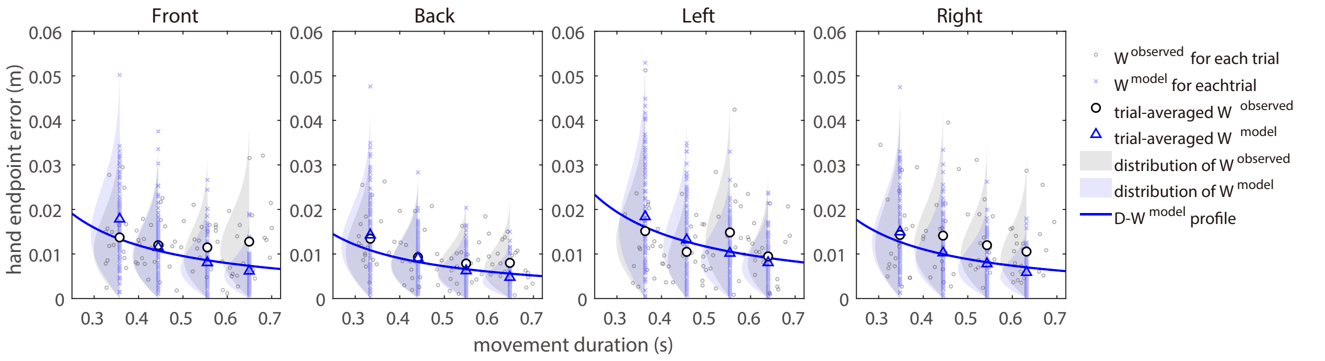


**(a)** *D–W* profile

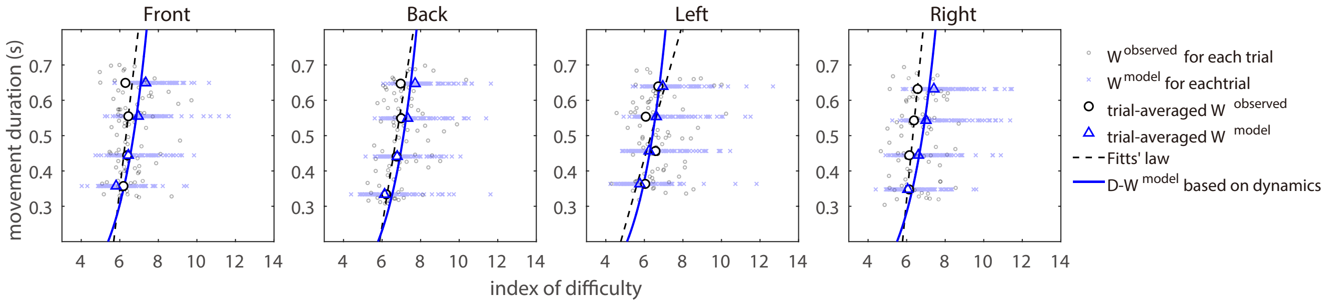


**(b)** $\log_2\left(\frac{1}{W}\right)$–$D$ profile (Fitts' law)

**Supplementary Figure S12.** Subject J

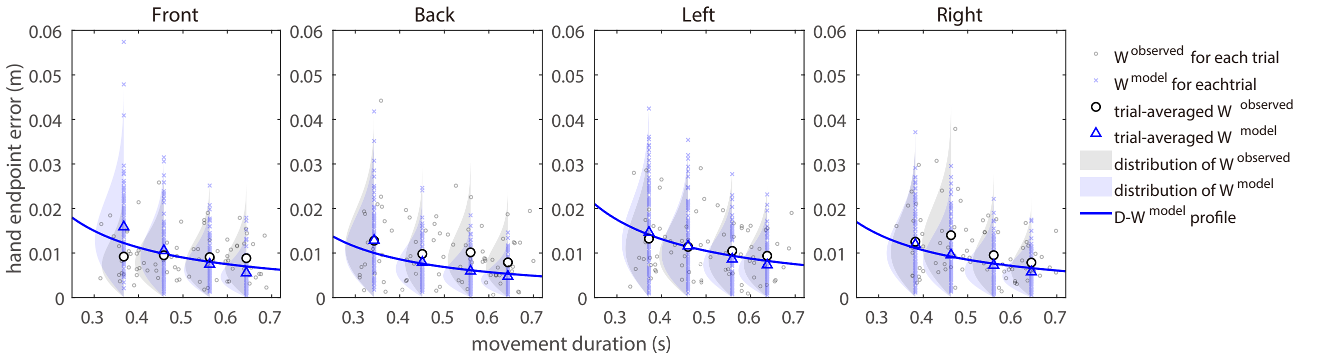


**(a)** *D–W* profile

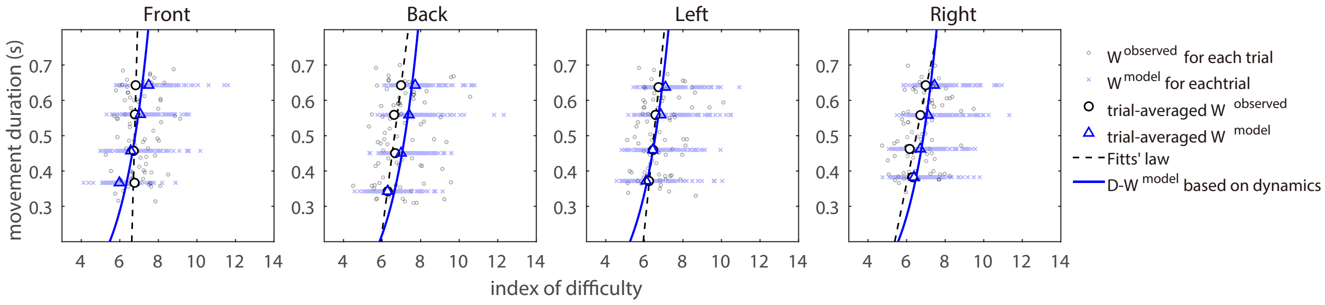


**(b)** $\log_2\left(\frac{1}{W}\right)$–$D$ profile (Fitts' law)

**Supplementary Figure S13.** Subject K

## Supplementary Discussion

### Comparison of results with and without Coriolis and centrifugal forces

Here, we present a comparison of the results obtained by the proposed model with and without Coriolis and centrifugal forces. Hereafter, the Coriolis and centrifugal forces are collectively referred to as nonlinear interference torque (NIT). Specifically, "with NIT" or "without NIT" are defined based on whether or not Coriolis and centrifugal forces are included in the time normalised torque $\tilde{\tau}(s)$ in Equations (13) and (14). Thus, in estimating the noise parameter $k_i$ and viscosity coefficient $B_{ij}$, in both cases, the calculation was performed using the torque with NIT.

We made an initial comparison using the data obtained from the experiment described in Methods (i.e., measuring the centre-out reaching movement in four directions: front, back, left and right at a 15-cm distance between the starting and target points). As shown in Supplementary Fig. S14, there was not much difference between with- and without-NIT cases. Supplementary Figure S15 shows normalised-time series of joint torques for each direction. In all directions, inertial and viscous forces dominate Coriolis and centrifugal forces. To the Left and Right in particular, Coriolis and centrifugal forces are nearly zero; in the Front and Back directions, the two forces cancel each other in the shoulder joint torque. These factors were presumably the reason for the lack of difference between the cases.

We then conducted an additional experiment with different starting and target postures to determine the results of rapid motion over a longer distance. Six healthy young adults (all males in the age range 23–24 y) participated; two of them (Subjects I and K) had also participated in the initial experiment. All subjects were right-handed. Informed consent agreements were obtained from all subjects, and the study was conducted according to the guidelines of the Declaration of Helsinki and approved by the Ethics Committee of Nagaoka University of Technology. Under the movement duration conditions of the original experiment, we increased the movement distance. The starting posture was defined as $\theta_1 = 106°$ and $\theta_2 = 87°$ and the target posture was defined as $\theta_1 = 14°$ and $\theta_2 = 71°$. The distance from the starting point to the target was 73.3 cm on average. Supplementary Table S3 lists the dynamics parameters and signal-dependent noise parameters estimated from each subject's data, and Supplementary Fig. S16 shows the results of the comparison for each subject. Aside from subject O, the trial-averaged values of the actual endpoint error (open circles) for each subject were better represented using the with-NIT model (blue triangles and solid lines). The with-NIT model results were closer to the actual data than even the distributions of endpoint errors. Interestingly, the results show that the use of NIT can improve the hand endpoint accuracy. These results demonstrate the importance of considering Coriolis and centrifugal forces in modelling very rapid reaching movements over long distances.

**Supplementary Table S3.** Physical parameters and noise parameters of the arm

| Subject | I | K | L | M | N | O | Mean ± SD* |
|---|---|---|---|---|---|---|---|
| $L_1$ (m) | 0.283 | 0.306 | 0.268 | 0.308 | 0.290 | 0.299 | 0.292±0.015 |
| $L_2$ (m) | 0.350 | 0.325 | 0.318 | 0.328 | 0.304 | 0.321 | 0.324±0.015 |
| $S_1$ (m) | 0.106 | 0.116 | 0.100 | 0.116 | 0.109 | 0.113 | 0.110±0.006 |
| $S_2$ (m) | 0.170 | 0.159 | 0.156 | 0.161 | 0.150 | 0.157 | 0.159±0.007 |
| $m_1$ (kg) | 1.402 | 1.528 | 1.320 | 1.538 | 1.438 | 1.490 | 1.453±0.083 |
| $m_2$ (kg) | 1.869 | 1.793 | 1.774 | 1.803 | 1.733 | 1.782 | 1.792±0.045 |
| $I_1$ ($\mathrm{kg \cdot m^2}$) | 0.024 | 0.031 | 0.020 | 0.032 | 0.026 | 0.029 | 0.027±0.005 |
| $I_2$ ($\mathrm{kg \cdot m^2}$) | 0.049 | 0.040 | 0.038 | 0.041 | 0.033 | 0.039 | 0.040±0.005 |
| $B_{11}$ ($\mathrm{kg \cdot m^2/s}$) | 1.015 | 1.142 | 1.010 | 1.138 | 1.050 | 1.099 | 1.076±0.059 |
| $B_{12}$ ($\mathrm{kg \cdot m^2/s}$) | 0.227 | 0.231 | 0.225 | 0.236 | 0.225 | 0.231 | 0.229±0.004 |
| $B_{21}$ ($\mathrm{kg \cdot m^2/s}$) | 0.227 | 0.231 | 0.225 | 0.236 | 0.225 | 0.231 | 0.229±0.004 |
| $B_{22}$ ($\mathrm{kg \cdot m^2/s}$) | 1.016 | 1.034 | 1.004 | 1.060 | 1.005 | 1.037 | 1.026±0.022 |
| $k_1$ | 0.300 | 0.265 | 0.250 | 0.286 | 0.330 | 0.393 | 0.304±0.052 |
| $k_2$ | 2.250 | 1.597 | 3.500 | 2.248 | 2.355 | 3.050 | 2.500±0.673 |

* SD: standard deviation across subjects.

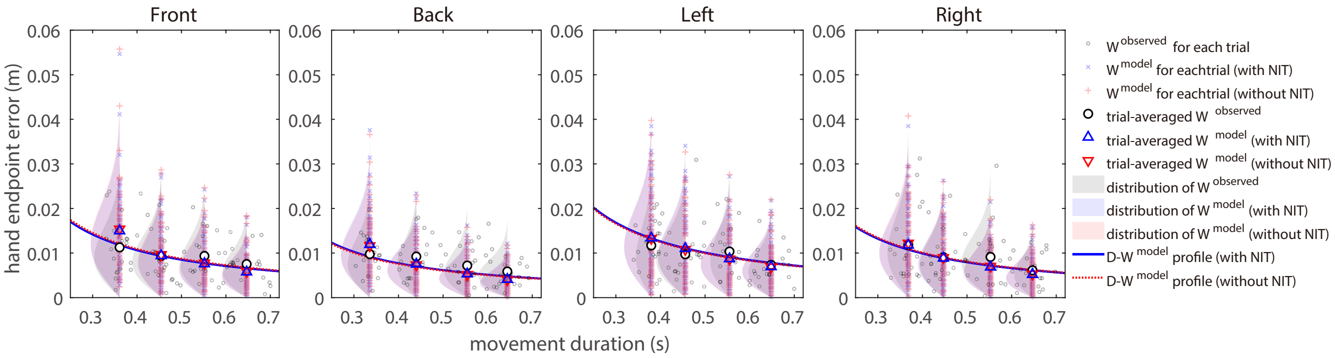


**Supplementary Figure S14.** Comparison with and without NIT of a representative subject (A) in the initial experiment.

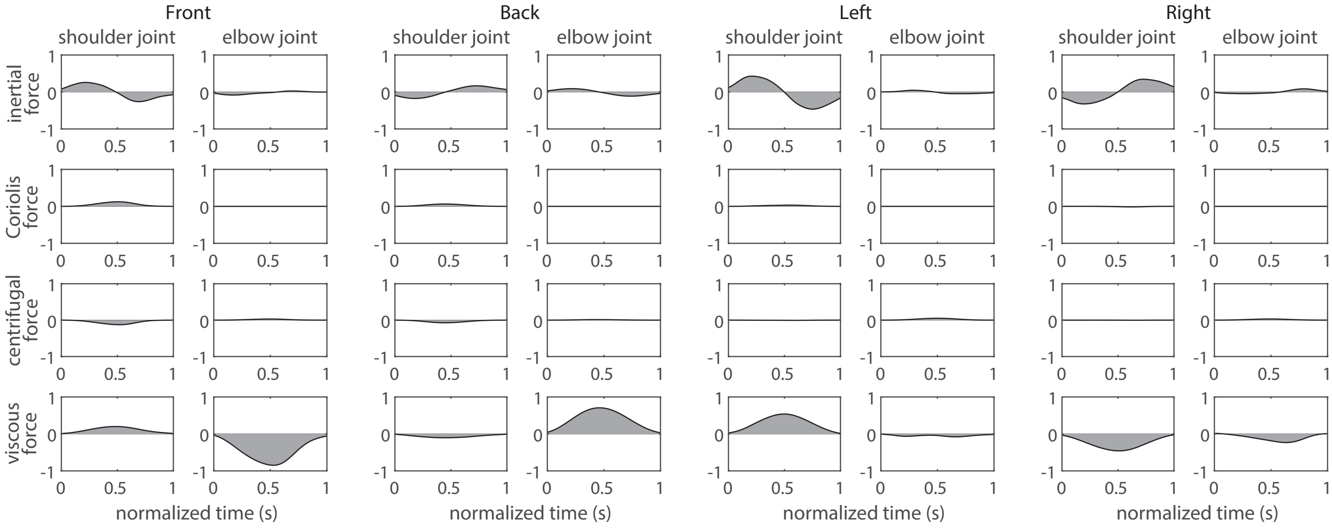


**Supplementary Figure S15.** Time-normalised series of inertial, Coriolis, centrifugal, and viscous forces for each direction of a representative subject (A) in the initial experiment.

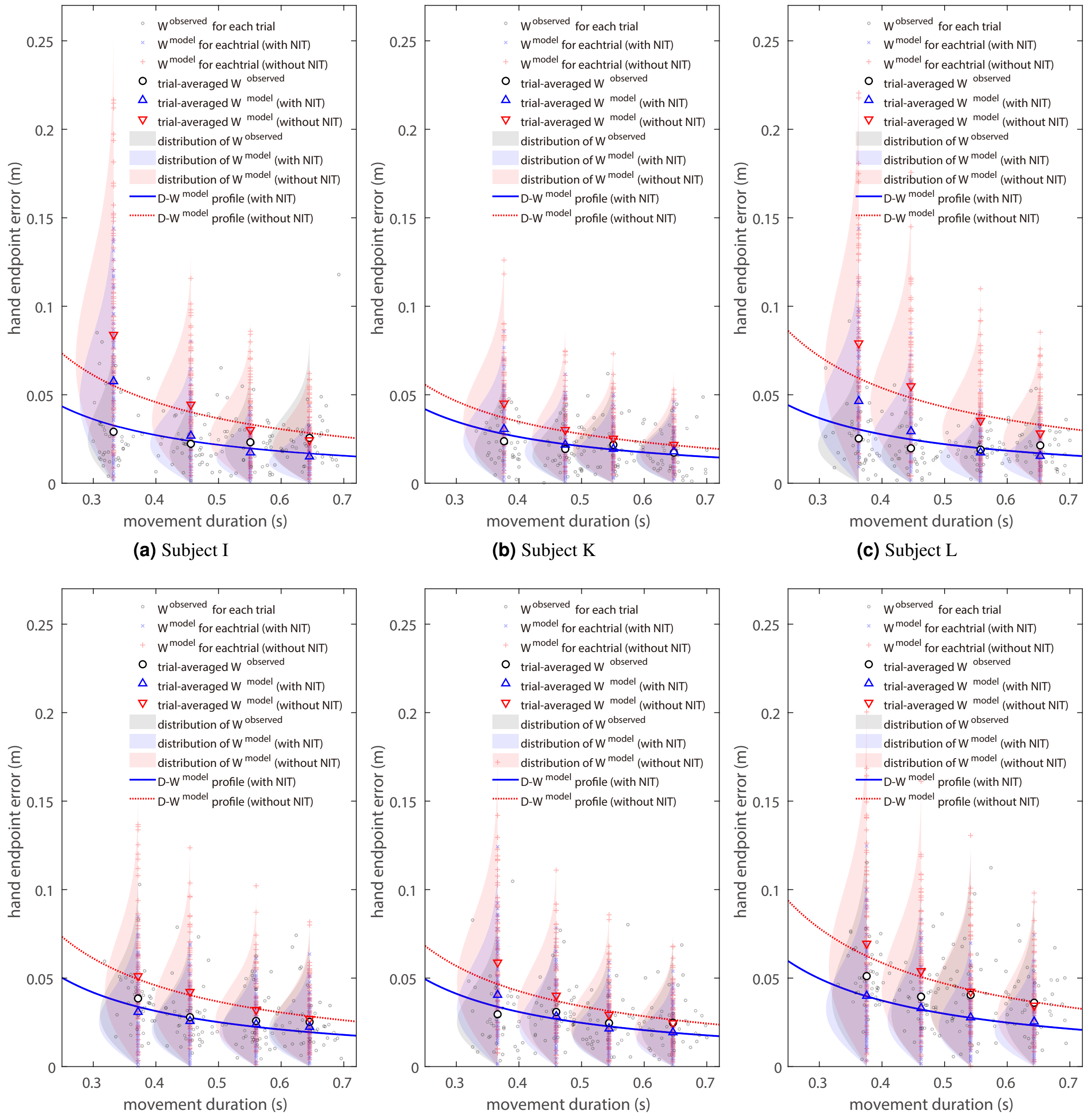


**(a)** Subject I **(b)** Subject K **(c)** Subject L

**(d)** Subject M **(e)** Subject N **(f)** Subject O

**Supplementary Figure S16.** Comparison with and without NIT of each subject in the additional experiment.
